# Comprehensive mineralogical and physicochemical characterization of recent sapropels from Romanian saline lakes for potential use in pelotherapy

**DOI:** 10.1038/s41598-021-97904-1

**Published:** 2021-09-20

**Authors:** Andreea Baricz, Erika A. Levei, Marin Șenilă, Simona Cîntă Pînzaru, Mihaela Aluaş, Adriana Vulpoi, Claudiu Filip, Carmen Tripon, Dorin Dădârlat, Doriana M. Buda, Francisc V. Dulf, Adela Pintea, Adorján Cristea, Vasile Muntean, Zsolt G. Keresztes, Mircea Alexe, Horia Leonard Banciu

**Affiliations:** 1 Department of Molecular Biology and Biotechnology, Faculty of Biology and Geology, Babeş-Bolyai University, 5-7 Clinicilor Str., 400006 Cluj-Napoca, Romania; 2 INCDO-INOE 2000, Research Institute for Analytical Instrumentation, 67 Donath Str., 400293 Cluj-Napoca, Romania; 3 Faculty of Physics, Babeş-Bolyai University, 1 M. Kogalniceanu Str., 400084 Cluj-Napoca, Romania; 4 Institute for Interdisciplinary Research in Bio-Nano-Sciences, Babeş-Bolyai University, Cluj-Napoca, Romania; 5 National Institute for Research and Development of Isotopic and Molecular Technologies, 67-103 Donath Str., 400293 Cluj-Napoca, Romania; 6 Centre for Systems Biology, Biodiversity and Bioresources, Babeş-Bolyai University, 400006 Cluj-Napoca, Romania; 7 Department of Engineering and Environmental Protection, University of Agricultural Sciences and Veterinary Medicine Cluj-Napoca, 400372 Cluj-Napoca, Romania; 8 Department of Chemistry and Biochemistry, University of Agricultural Sciences and Veterinary Medicine Cluj-Napoca, 400372 Cluj-Napoca, Romania; 9 Department of Physical and Technical Geography, Faculty of Geography, Babeș-Bolyai University, 5-7 Clinicilor Str., 400006 Cluj-Napoca, Romania

**Keywords:** Stable isotope analysis, Biogeochemistry, Environmental sciences, Limnology, Mineralogy, Geochemistry

## Abstract

In this study we aimed to compare the mineralogical, thermal, physicochemical, and biological characteristics of recent organic carbon-rich sediments (‘sapropels’) from three geographically distant Romanian lakes (Tekirghiol and Amara, SE Romania, and Ursu, Central Romania) with distinct hydrogeochemical origins, presently used for pelotherapy. The investigated lakes were classified as inland brackish Na-Cl-sulfated type (Amara), coastal moderately saline and inland hypersaline Na-Cl types (Tekirghiol and Ursu, respectively). The settled organic matter is largely composed of photosynthetic pigments derived from autochthonous phytoplankton. Kerogen was identified in the sapropel of coastal Tekirghiol Lake suggesting its incipient maturation stage. The mineral composition was fairly similar in all sapropels and mainly consisted of quartz, calcite, and aragonite. Smectite, illite, mixed layer smectite/illite appeared as major clay components. Potentially toxic elements were found in low concentrations. The physical properties (i.e., specific heat, thermal conductivity and retentivity) and cation exchange capacity are comparable to other peloids used for therapy. This study is the first comprehensive multi-approached investigation of the geochemical nature of recent sapropels in Romanian saline lakes and thus contributes to expanding our knowledge on the origin and physicochemical qualities of organic matter-rich peloids with therapeutic uses.

## Introduction

Maturated muds termed ‘peloids’ are composed of fine-grained minerals and/or organic matter derived from biological metabolic activity, mixed with sea water, salt water, or spring derived mineral-medicinal waters, and are widely used, in different cultures, for therapeutic purposes^1–7^. Sapropels are organic rich sediments (> 2% organic carbon) formed in stagnant water basins, mainly inland or coastal saline lakes, with high primary productivity and oxygen-depleted bottom water^8^. Contemporary sapropels (i.e., high saline modern sediments) undergo natural maturation to form natural peloids used as healing muds or muddy suspensions for curative purposes, at their occurrence sites^3^. Organic-rich peloids from saline lakes are used together with mineral-rich brine water in treatments associated with musculoskeletal and nervous system disorders^8,9^. Naturally formed peloids have been investigated in marine areas from Croatia^10–12^, the Dead Sea and Crimean saline lakes^8^.

Knowledge of the therapeutic value of Romanian saline lakes extends over many centuries, as early as Dacian–Roman times^13^ and numerous saline lakes of varying hydrogeochemical nature are found in the Transylvanian (Central Romania) and Dacian (South-Eastern) Basins^14^.

Tekirghiol, Amara and Ursu lakes are naturally-formed and defined by markedly different salinities, hydric regimes, trophic state, local climate and genesis mechanisms. While Amara and Tekirghiol have been sparsely explored for their chemistry and biology, Ursu Lake has been intensively studied since the beginning of the twentieth century, including recent snapshot on chemistry and associated biology of sapropels^7^. Harboring large deposits of sapropels, Tekirghiol, Amara and Ursu lakes are readily exploited for therapeutic purposes^13^. Natural sapropels (and water) from these saline lakes have been used as treatment for musculoskeletal disorders, gynecological, endocrine, dermatological, and hepatobiliary diseases^2,9^.

**Figure 1:**
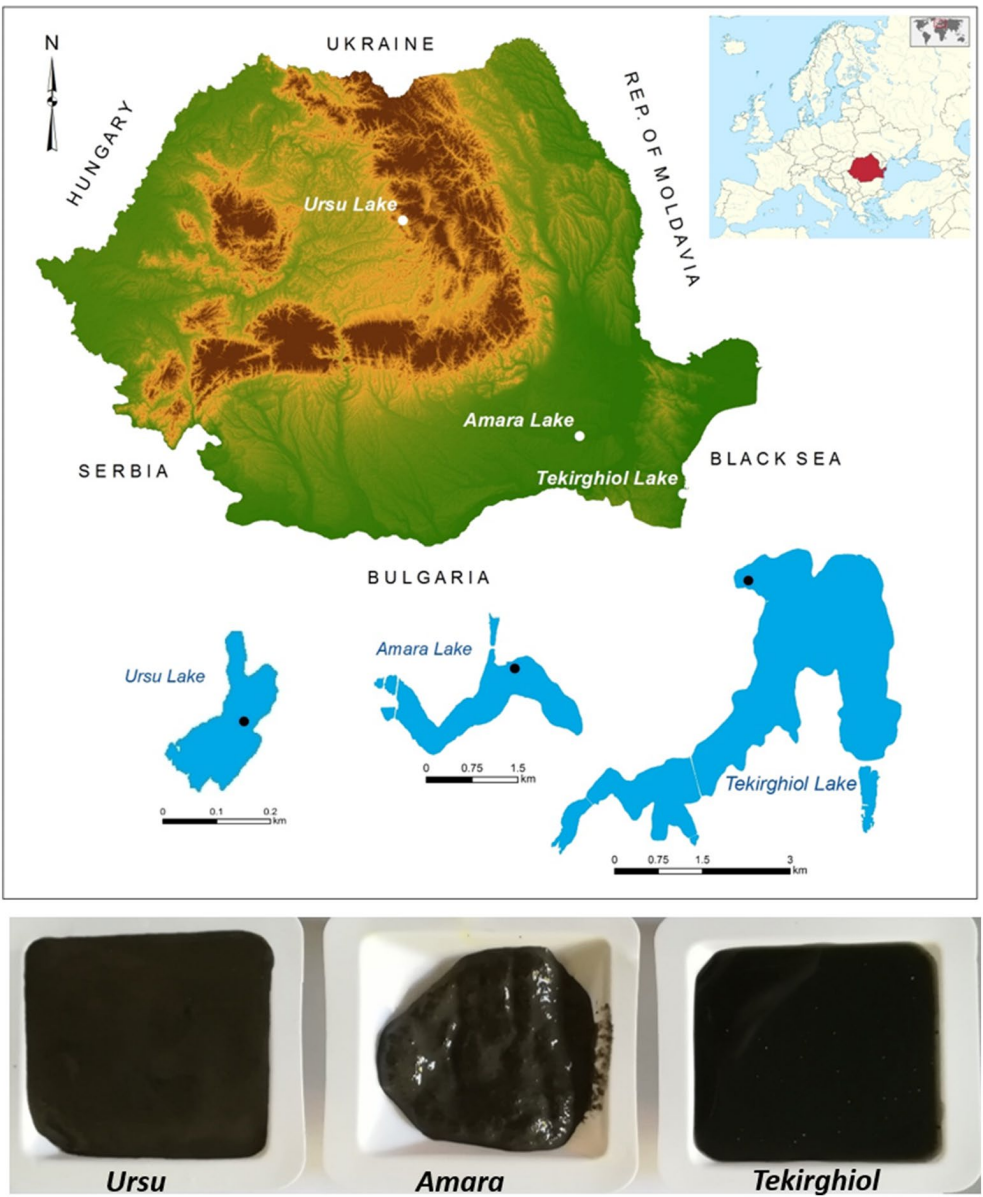
Geographic location and morphometric details of peloidogenous Ursu, Amara and Tekirghiol lakes, and the macroscopic aspect of the recent, dark-colored sapropels with curative use. The sampling points are indicated by black dots within the contour of each tested lake. ArcGIS 10.2 software was used to create the map (Environmental Systems Research Institute, USA, http://www.esri.com). The map of Romania is based on the EU-DEM dataset (European Digital Elevation Model), and the contours of the lakes were obtained by digitization (Google Maps).

Several studies on pelotherapy suggest that the physicochemical properties controlled by the mineral composition determine the suitability of sapropels to be used for therapeutic purposes. The clay mineral content (mainly smectite) is relevant to therapy, and it also determines other properties, i.e., high water absorption, high specific surface area and cation exchange capacity (which provide high capacity to trap unwanted elements), and high specific heat^15^. Furthermore, the clay content influences adsorption of greases and toxins, and as well heat retention and malleability^16^. Natural sediments, however are rarely composed of pure clay, containing a variety of different minerals derived from the sedimentary environment. Moreover, carbonates present in the sapropels stimulate subcutaneous circulation and optimal stratification of the epidermis, while sulfur content of sapropels could influence the therapeutic action, as sulfur shows a keratolytic effect, analgesic influence on the pain receptors and inhibition of the immune response, and has a bactericidal and antifungal effect^4^. Tekirghiol, Amara and Ursu lakes (Fig. 1) are amidst the most important lakes of therapeutic use in Romania, as they possess the required characteristics for balneo- and pelotherapy, i.e., considerable water depth (> 0.5 m), thickness of black and/or grey organic muds around 0.01–1.2 m^17^. The modern sapropels of Romanian saline lakes have been investigated in recent times^18^ to assess the microbial diversity and functionality of inhabiting microorganisms, yet the complex interplay of mineralogical, physical and chemical factors affecting sapropel properties is poorly understood. Our main aim is to gain insights into the mineralogy, chemistry and thermo-physical properties of sapropels from three lakes with distinct origin and contrasting salinities, and to evaluate their characteristics in respect to their therapeutic potential.

## Results

### Mineralogy and thermal properties

**Figure 2:**
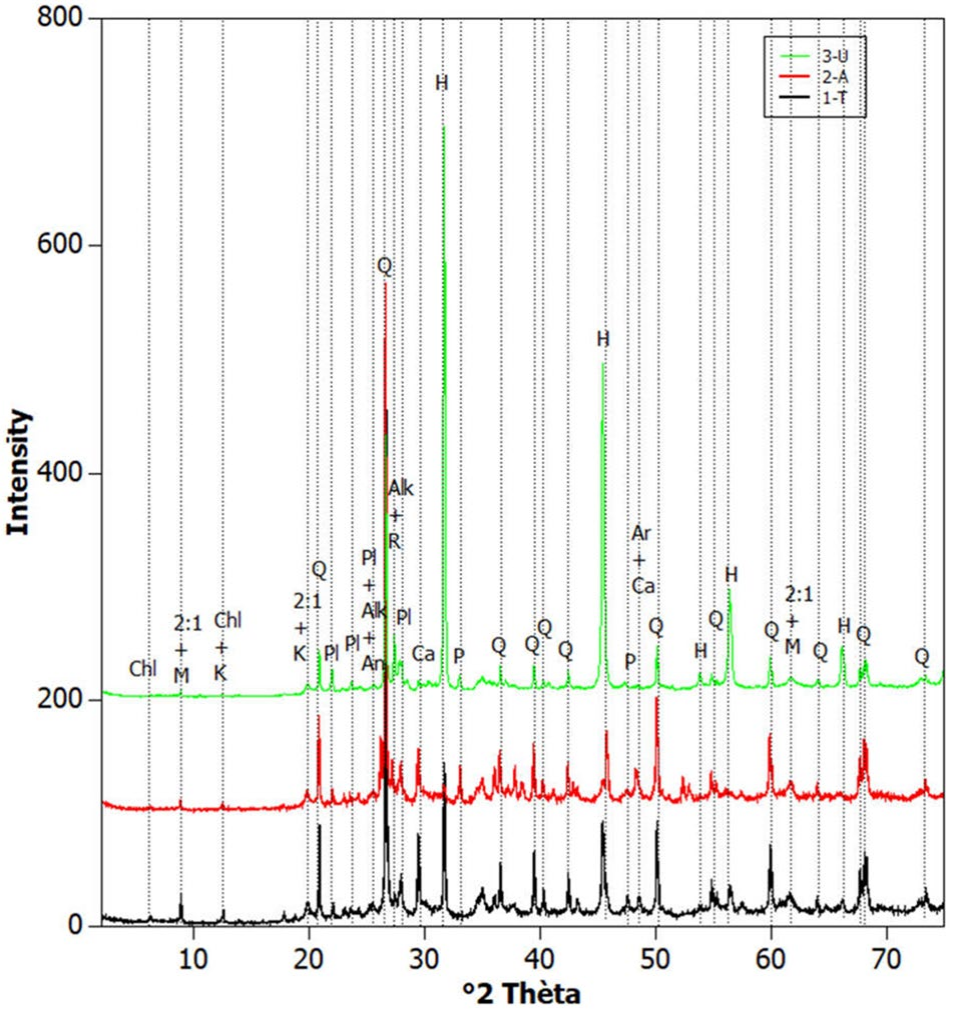
X-ray diffraction patterns on the raw mud samples (upper image) collected from the three lakes. The main minerals that contribute to the most important reflections are indicated. Chl: Chlorite, M: Muscovite, K: Kaolinite Group minerals, Q: Quartz, A: Anatase, 2:1: 2:1 phyllosilicate (e.g., illite and smectite), Ca: Calcite, Pl: Plagioclase/Albite/Andesine, R: Rutile, P: Pyrite, Ar: Aragonite, H: Halite.

**Figure 3:**
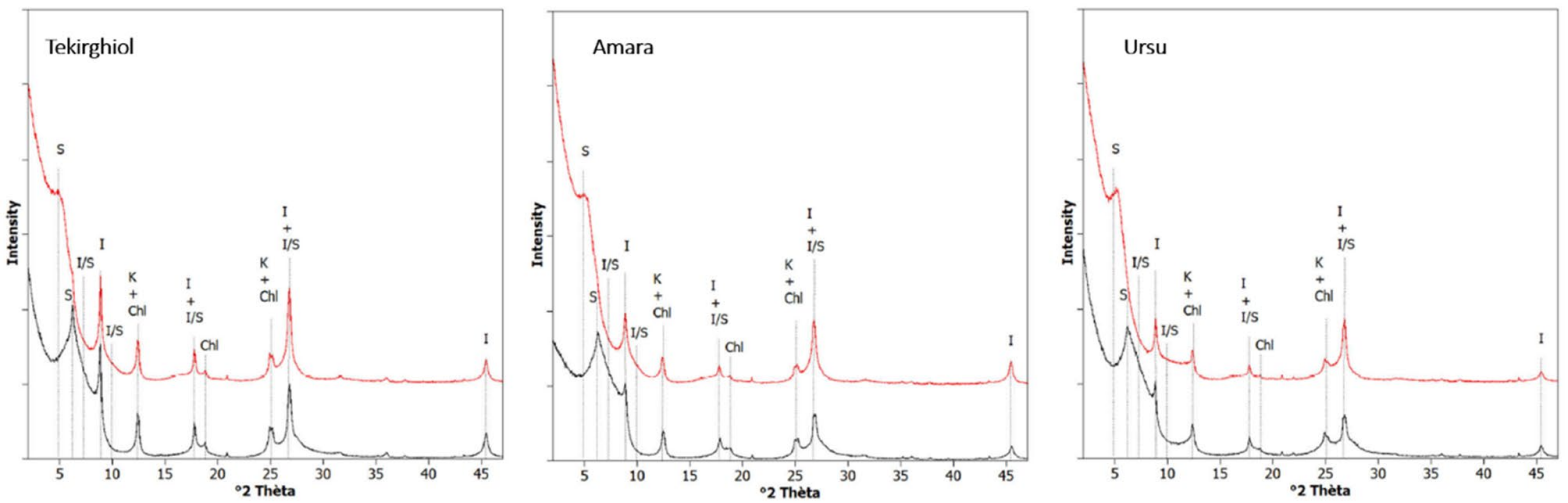
Diffraction patterns of air dried (red) and ethylene glycolated (black) oriented clay fractions in the three sapropels. The most important reflections are labeled: K—Kaolinite; I—Illite; I/S—Illite/Smectite; S—Smectite; Chl—Chlorite.

The bulk mineral composition of sapropels is detailed in Table 1. The XRD analysis indicates that Amara and Tekirghiol sapropels are enriched in silicates, i.e., quartz (30.8% and 29.1% respectively), plagioclase-albite (10.1% and 8.9%), carbonates, mainly calcite (6.8%) and aragonite (13.1%) in Amara, and calcite (8.7%) in Tekirghiol (Fig. 2). By contrast, Ursu sapropel contains lower concentrations of silicates, mainly quartz (15.4%), plagioclase (5.5% albite and 8% andesine), sulfides, i.e., pyrite (1.5%) and is enriched in halite (34.5%). The major clay components in the sapropels were 2:1 dioactahedral and 2:1 trioctahedral clays, representing 28.9%, 23.6% and 20.8% of clay minerals in Tekirghiol, Amara and Ursu samples, respectively. Muscovite was detected in similar concentrations in Tekirghiol (4.5%) and Amara (4.2%). Quantitative mineralogical clay composition of the fraction < 2 μm shows that these sapropels are defined by the presence of illite, smectite and interstratified illite/smectite as major components (> 90% in each sample), and kaolinite and chlorite as minor fractions (Table 2; Fig. 3).

**Table 1:** Quantitative bulk mineralogical compositions of saline sapropels collected from Tekirghiol, Amara and Ursu lakes.

	Tekirghiol Lake		Amara Lake		Ursu Lake
**NON-CLAYS**					
**Silicates**					
Quartz	29.1	30.8		15.4	
Cristobalite	–	–		0.7	
Opal	–	–		2.5	
Alkalifeldspar	3.1	2.8		3.6	
Plagioclase-Albite	8.9	10.1		5.5	
Plagioclase-Andesine	–	–		8.0	
Amphibole	0.1	0.3		0.9	
Pyroxene	1.7	1.3		1.1	
**Carbonates**					
Calcite	8.7	6.8		1.2	
Aragonite	–	13.1		–	
Ankerite	1.0	1.1		0.2	
**Sulfides**					
Pyrite	0.2	0.2		1.5	
**Oxides**					
Anatase	0.5	0.3		0.3	
Rutile	0.3	0.2		0.2	
**Phosphates**					
Apatite	–	0.5		–	
**Salts**					
Halite	8.4	1.4		34.5	
**CLAYS**					
Muscovite	4.5	4.2		0.1	
2:1 dioactahedral clays	22.3	18.2		15.9	
2:1 trioctahedral clays	6.7	5.4		4.9	
Kaolinite	2.0	1.1		2.8	
Chlorite	2.7	2.1		0.7	

Values are given in weight percentages of the identified minerals.

**Table 2:** Quantitative mineralogical clay composition of the fraction < 2 μm of the samples (in weight percentages).

	Tekirghiol Lake	Amara Lake	Ursu Lake
Illite	37	24.9	18.5
Smectite	20.5	22.9	25.4
Interstratified Illite/Smectite R0 (60/40)	33.2	45.4	47.7
Chlorite	3.5	2.1	0.2
Kaolinite	5.8	4.7	8.2

### Differential thermal analysis and thermogravimetric analysis

The thermal behavior of sapropel samples is presented in Table 3. Total mass loss ranged from 21.84% (Tekirghiol) to 24.72% (Ursu). TGA shows the main mass loss at temperatures between 200 and 700 °C for Amara sapropel (16.02%) and between 700 and 1000 °C for Ursu sapropel (15.76%), while stepwise significant mass loss was observed for Tekirghiol sapropel at temperatures between 200 and 1000 °C (10.23% at 200–700 °C and 9.32% at 700–1000 °C).

**Table 3:** Mass loss determined by thermogravimetric analysis of sapropel samples.

Sapropel	Total mass loss	Partial mass loss	Partial mass loss	Partial mass loss
		< 200 °C	200–700 °C	700–1000 °C
Tekirghiol	21.84	2.29	10.23	9.32
Amara	23.32	2.24	16.02	5.06
Ursu	24.72	2.04	6.94	15.76

**Figure 4:**
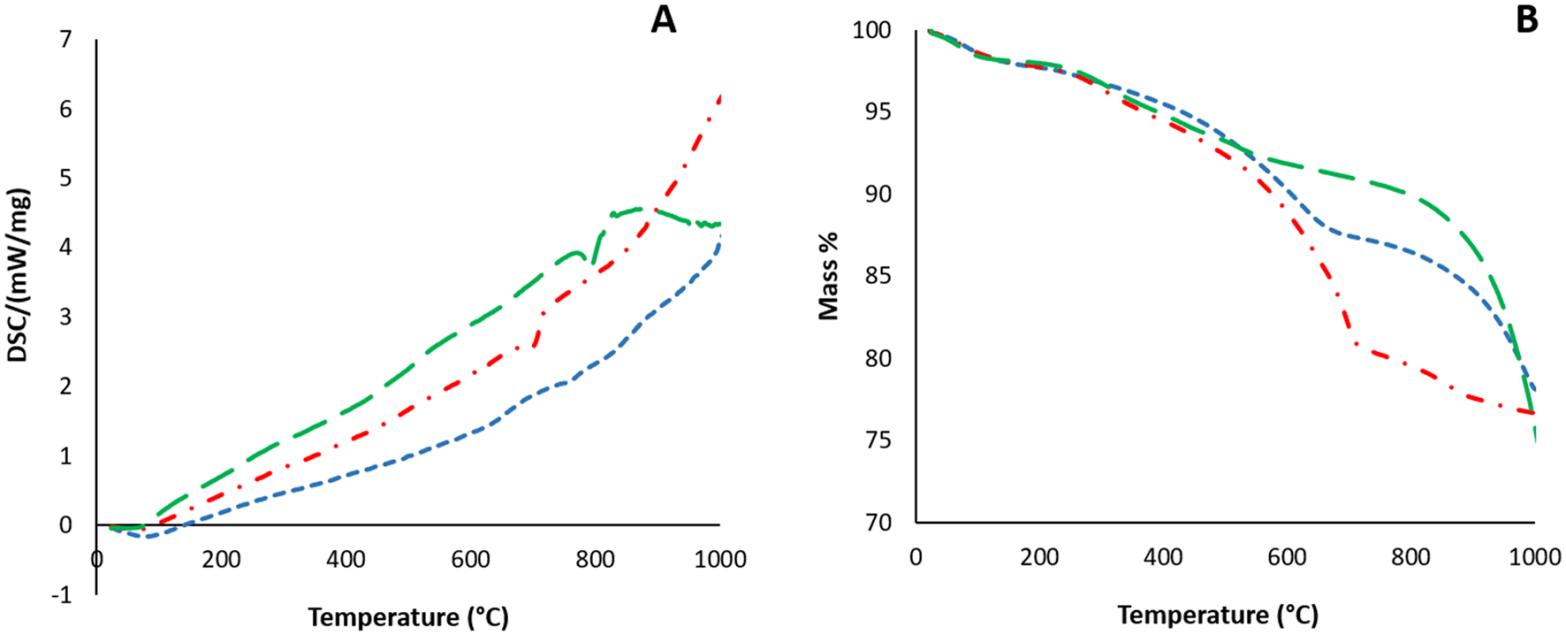
Thermal analysis of Tekirghiol (blue dashed line), Amara (red dashed line) and Ursu (green dashed line) sapropels by differential scanning calorimetry (**A**) and thermogravimetric analysis (**B**).

For all analyzed samples, the TGA/DSC curves (Fig. 4) indicate the removal of molecular water at lower temperatures (200 °C), followed by a second event causing substantial mass loss possibly due to dehydroxylation of illite and smectite, the main clay minerals in these sapropels. Decomposition of carbonates takes place above 650 °C (up to 900 °C), and is probably responsible for the considerable mass loss detected in Amara sapropel.

### Physical characteristics

Particle size analysis revealed that all three sapropels can be defined as sandy silts (Table 4). Tekirghiol sapropel was 74.4% silt, 13% clay and 12.7% sand, compared to Ursu sapropel with 65.9% silt, 9% clay and 25% sand. The Amara sapropel contained the highest concentration of sand particles (35%), lowest concentration of clay (7.2%) and silt (57%). In Tekirghiol and Ursu samples the mud fraction (silt and clay) was considerably greater (87.5% and 74.9%, respectively) than the sand fraction as compared to Amara sample (64.4%). Similar CEC values were determined for Tekirghiol (27.2 cmol(+)/kg) and Amara (24 cmol(+)/kg). The sapropel from Ursu Lake featured highest density, humidity and cation exchange capacity, and lowest specific surface area (Table 4) among all analyzed sediments. To verify the influence of salt contents on the SSA, we performed the measurements both in raw (salted) and desalted sapropels. The highest increase of SSA after desalinization (more than 3 times, from 2.77 to 8.7 m^2^ g^−1^) was observed for hypersaline Ursu Lake sapropel. In Amara and Tekirghiol sediments, the SSA increase after desalinization was 1.6 times (from 8.3 to 13.7 m^2^ g^−1^) and 1.3 times (from 12.63 to 16.53 m^2^ g^−1^), respectively. The total pore volumes, which could be measured only after desalinization, showed similar values, ranging from 0.034 ml g^−1^ in Ursu sapropel, to 0.031 ml g^−1^ in Amara and 0.030 ml g^−1^ in Tekirghiol. These findings suggest enhanced water entrapment corroborated by larger total pore volume and enrichment in NaCl (Table 5) which might explain the higher humidity of Ursu sapropel as compared to the other tested muds.

**Table 4:** Physical and physicochemical properties of saline sapropels collected from Tekirghiol, Amara and Ursu lakes.

Parameter	Tekirghiol Lake	Amara Lake	Ursu Lake
Density (kg m^−3^)	1148.56	1168.30	1177.22
Humidity (as water content of wet sediment, %)	66.96	34.20	70.04
Specific surface area in native sapropel (SSA, m^2^ g^−1^)	12.63	8.30	2.77
Specific surface area in desalted sapropel (SSA, m^2^ g^−1^)	16.53	13.70	8.70
Total cation exchange capacity (CEC, cmol(+)/kg)	27.2	24	38.4
Thermal conductivity (W/m·K)	0.65 ± 0.04	0.56 ± 0.03	0.53 ± 0.03
Thermal retentivity (× 10^6^ s m^−2^)	4.09 ± 0.13	4.62 ± 0.10	6.35 ± 0.17
Thermal diffusivity (× 10^7^ m^2^ s^−1^)	2.46 ± 0.03	2.17 ± 0.03	1.57 ± 0.02
Specific heat (× 10^3^ J kg^−1^ K^−1^)	2.31 ± 0.16	2.21 ± 0.13	2.86 ± 0.20
Granulometry—Sand (%, 63–2000 µm)	12.7	35.6	25.1
Granulometry—Silt (%, 2–63 µm)	74.4	57.2	65.9
Granulometry—Clay (%, < 2 µm)	13.0	7.2	9.0

**Table 5:** Chemical and nutrient composition (average ± standard deviation of three replicates) of the sediments collected from Tekirghiol, Amara and Ursu lakes.

Compound (mg kg^−1^)	Tekirghiol Lake	Amara Lake	Ursu Lake
**Chemical composition**			
Na^+^ (Total)	17,367 ± 624	8967 ± 348	271,333 ± 9855
K^+^ (Total)	6267 ± 220	4900 ± 196	720 ± 28
Ca^2+^ (Total)	56,067 ± 1842	97,667 ± 3210	13,933 ± 557
Mg^2+^ (Total)	15,017 ± 604	14,373 ± 535	2470 ± 99
Cl^−^ (Water sol.)	27,700 ± 889	11,200 ± 228	410,000 ± 9400
Fe (Total)	17,910 ± 716	17,997 ± 519	6867 ± 274
Mn (Total)	195 ± 12	575 ± 32	216 ± 12
Cu (Total)	26.1 ± 1.56	24.6 ± 1.4	13.8 ± 0.8
Ba (Total)	76 ± 4.6	87 ± 5	11 ± 1
Al (Total)	18,290 ± 1070	16,947 ± 1010	9150 ± 349
Zn (Total)	106.5 ± 6.4	76 ± 6	128.1 ± 7.7
Pb (Total)	11.5 ± 0.7	7.5 ± 0.5	14.5 ± 0.9
Cr (Total)	23.4 ± 1.4	19.7 ± 1.1	4.3 ± 0.4
Sr (Total)	124 ± 8	49 ± 3	43 ± 2
Ni (Total)	17.3 ± 1.1	18.7 ± 1.2	4.7 ± 0.3
Co (Total)	5.1 ± 0.5	5.8 ± 0.5	1.4 ± 0.1
As (Total)	4.97 ± 0.45	8.16 ± 0.66	3.31 ± 0.24
Cd (total)	0.80 ± 0.1	0.81 ± 0.07	1.12 ± 0.10
**Water soluble nutrient composition**			
DTN	172 ± 12	107 ± 7	247 ± 16
NO_3_^−^	180 ± 9	52.5 ± 3.5	< 2.0
NO_2_^−^	< 0.5	< 0.5	< 0.5
N–NH_4_	125 ± 8	75 ± 5	240 ± 14
SO_4_^2−^	250 ± 14	13,240 ± 850	280 ± 18
Sulfides	20.6 ± 1.3	18.5 ± 1.2	20.2 ± 1.4
Total P	1.90 ± 0.11	2.33 ± 0.17	2.85 ± 0.18
DTC	1051 ± 65	1021 ± 62	1152 ± 76
DIC	576 ± 38	616 ± 44	314 ± 20
DOC (= DC-DIC)	475 ± 30	405 ± 26	838 ± 52
C/N ratio	6.11 ± 0.44	9.54 ± 0.62	4.66 ± 0.33

Total—sediments were dried and extracted with *aqua regia*, therefore values are meant for total mineral contents; Water sol.—sediments were levigated 1/10 in water, therefore values are meant for water soluble minerals.

### Chemical composition of sapropels

**Figure 5:**
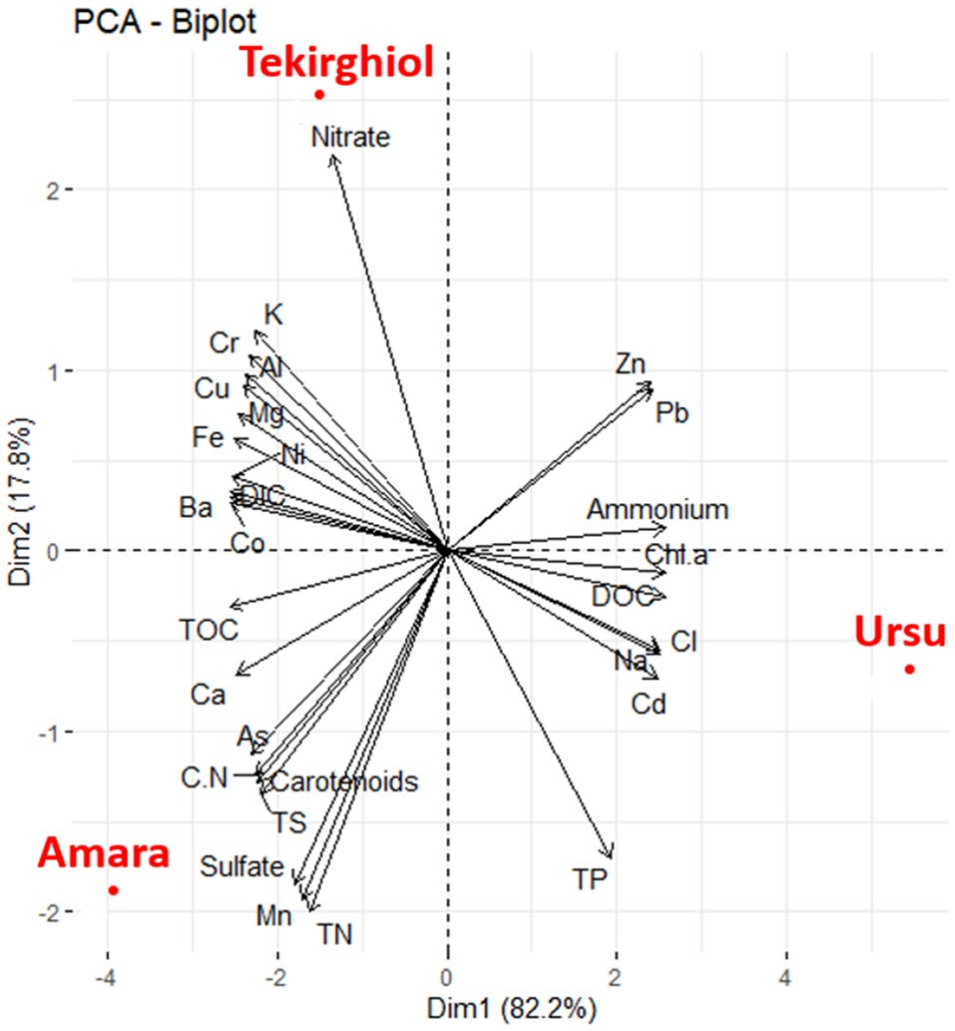
PCA (Principal component analysis) plot indicating the main physicochemical parameters that contribute to sapropel differentiation.

The overall chemistry of the explored sapropels and overlying water is highlighted in Tables 5, 6 and Supplementary Table 1, respectively. The salt contents and pH ranged from sulfate-enriched, low-saline and slightly alkaline (Amara), to chloride-dominated, moderately saline and pH-neutral (Tekirghiol) or hypersaline and slightly acidic (Ursu). PCA (Principal Component Analysis) further differentiates the three lakes sapropels, as Tekirghiol seems to be defined by the inorganic carbon and metal concentrations (i.e., Al, Mg, Fe, K), Amara by higher calcium and sulfates contents (Fig. 5), and hypersaline Ursu sediment by Na^+^, Cl^−^, dissolved organic carbon (DOC) and phosphorous. Except for Sr, detected in higher concentrations (124 mg kg^−1^) mainly in Tekirghiol sapropel compared to Amara (47 mg kg^−1^) and Ursu (43 mg kg^−1^), potentially toxic elements such as As, Co, Cr, Cd, Cu, Ni, Pb, are present in low concentrations (Table 4). The predominant rare earth elements shared by all samples are cerium and neodymium (~ 20 and ~ 10 mg kg^−1^ in Tekirghiol and Amara; ~ 5 and ~ 3 mg kg^−1^ in Ursu Llake, respectively). Ursu Lake sediment has substantially (i.e., ~ fourfold) lower lanthanide concentrations that Tekirghiol and Amara (Supplementary Table 2).

**Table 6:** Chlorophyll, organic matter, elemental contents and stable isotope composition (average ± standard deviation of three replicates) of bulk sediments of Tekirghiol, Amara and Ursu lakes.

Parameter (fraction of material)	Tekirghiol Lake	Amara Lake	Ursu Lake
pH (pore water)	7.38 ± 0.22	8.03 ± 0.24	6.55 ± 0.18
Total chlorophyll (µg L^−1^) (in pore water)	3011.52	2348.88	5490.86
Total carotenoids (µg L^−1^) (in pore water)	4072	4924	3746
Total protein (% wet sediment)	1.80 ± 0.10	0.64 ± 0.06	1.50 ± 0.09
Total organic C (TOC, % dry sediment)	2.09 ± 0.19	3.21 ± 0.28	3.24 ± 0.26
Total N (% dry sediment)	0.25 ± 0.02	0.35 ± 0.03	0.26 ± 0.02
Total S (% dry sediment)	0.87 ± 0.06	1.40 ± 0.10	0.69 ± 0.06
δ^13^C_OC_ (‰ dry sediment)	− 23.87	− 27.77	− 27.70
δ^13^C_DIC_ (‰ dry sediment)	− 6.18	− 2.95	− 7.38
δ^18^O (‰ sediment carbonates)	− 5.64	− 2.98	− 9.68
δ^15^N bulk (‰ dry sediment)	12.38	5.13	4.98
δ^34^S bulk (‰ dry sediment)	− 2.26	− 20.17	0.11
δ^34^S sulfides (‰ pore water)	7.74	8.55	− 5.21
δ^34^S sulfates (‰ pore water)	37.23	18.17	28.42
Total C/N (% w/w) (from combusted sediments)	8.49 ± 0.67	7.35 ± 0.62	5.61 ± 0.50

### Raman spectroscopy

**Figure 6:**
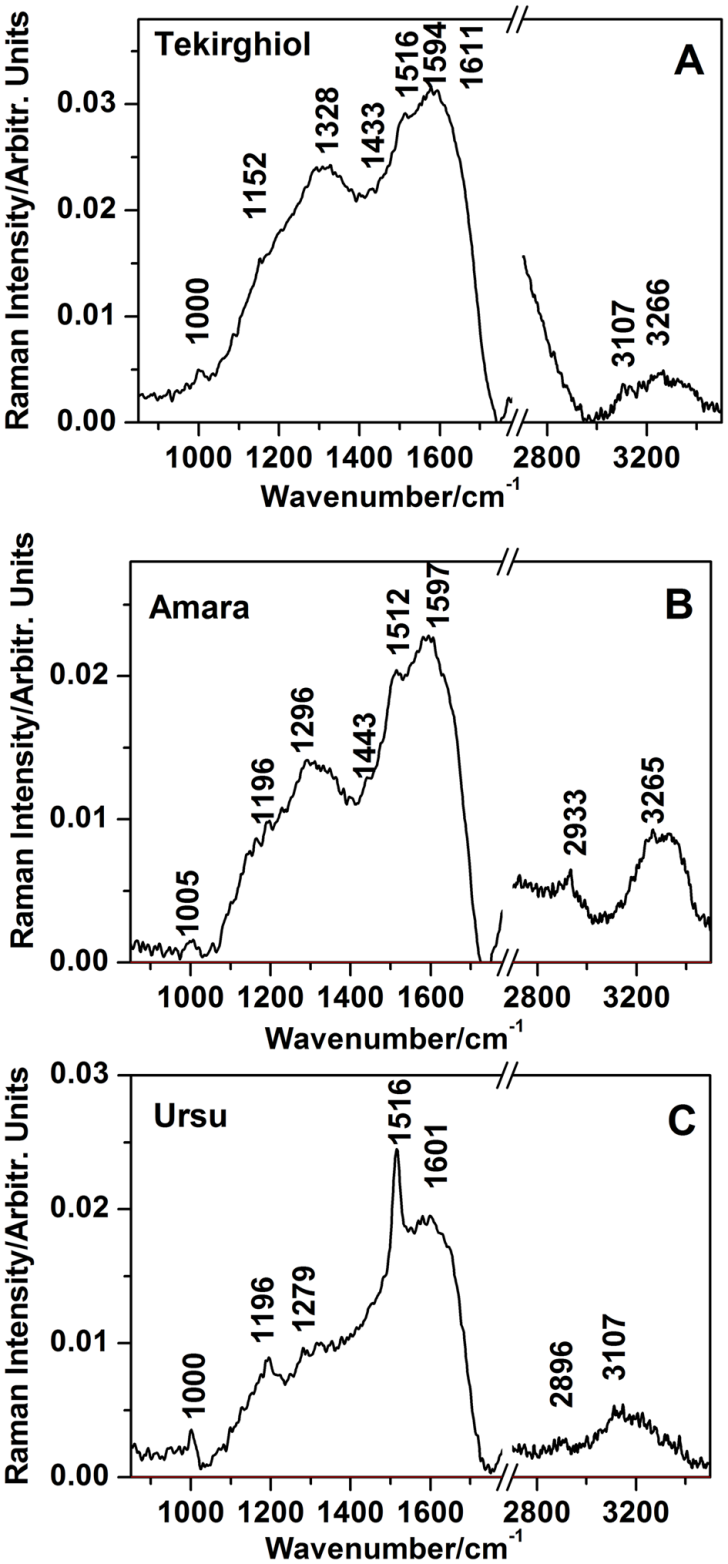
FT-Raman spectra of the bulk sapropels from Tekirghiol (**A**), Amara (**B**) and Ursu lakes (**C**). Note the highest carotenoid band at 1516 cm^−1^ in Ursu and completely different spectral profile comprising overlapped contributions from organic material (amorphous carbon, trace of aliphatic and aromatic compounds, photosynthetic pigments). Excitation: 1064 nm. Spectra were background subtracted.

FT-Raman spectra of the bulk samples (Fig. 6) showed main bands characteristic to the C=C skeletal stretching mode of carotenoids (1512–1516 cm^−1^), with highest intensity in Ursu sapropel. Substantial presence of methylated compounds was indicated by bands at 1433–1445 cm^−1^ and 2933 cm^−1^. The broaden band at around 1600 cm^−1^ suggested an intricate overlap of organic compounds. For Tekirghiol and Amara sapropels confocal Raman spectra (532 nm excitation) pointed on the presence of β-carotene most probably originating from cyanobacteria (Supplementary Figures S1 and S2). The main bands specific to carotenoids were obtained from “dark green” spots of about 1–3 μm and recorded at 1518–1520 (C=C), 1156 (C–C) and 1004–1006 cm^−1^ (C–CH_3_). Noteworthy, in Tekirghiol sample, the signal assigned to kerogen has been recorded from multiple “black” spots and recognized according to the characteristic D and G bands at 1360 and 1561–1601 cm^−1^ respectively (Supplementary Figure S1). Additional spurious bands were associated with traces of organic matter (amide of proteins at 1657 cm^−1^, S–S and C–S at 561 and 645 cm^−1^ respectively).

Two types of carotenoid signatures were distinguishable in Ursu Lake sapropel, tentatively associated with the main stretching modes of β-carotene (1516 cm^−1^) in Cyanobacteria and Archaea and fucoxanthin (1528 cm^−1^) in diatoms (Supplementary Figure S6). The SERS test revealed only the enhancement of the carotenoids resonance Raman bands and a considerable decrease of the background intensity level (Supplementary Fig. S3, B). Contrary to the expectations, humic substances were not detectable.

### FT-IR spectroscopy and SEM

SEM–EDX analyses of Amara and Tekirghiol sapropels showed substantial presence of O, Al, Si and metals like Mg, Fe, K (Supplementary Figure S4), and high content of Na and Cl (21.59% and 31.38% relative concentration, respectively) in Ursu Lake sapropel, demonstrating the substantial discrepancy in salinity compared to Tekirghiol (Na—2.83%, Cl—2.74%) and Amara sapropels (Na—1.6%, Cl—0.69%). The Amara sapropel appeared as the most enriched in Ca^2+^ followed by Tekirghiol (6.91%) and Ursu sapropels (1.69%). Trace amounts of titanium were detected in all samples, while trace amounts of fluorine were detected only in Tekirghiol and Ursu. Amara Lake sapropel shows higher S content (1.49%) compared to Tekirghiol (0.88%) and Ursu sapropels (0.93%). S-rich inorganic complexes were detected by EDX elemental mapping and FT-IR spectroscopy analyses in Amara and Ursu samples (Supplementary Figure S5) whereas FT-IR spectra of Amara sample showed distinguishable absorption bands associated to –SH (at 2590 cm^−1^), O=S=O (1140 cm^−1^) and S–O stretching modes (600–700 cm^−1^)^19^ (Supplementary Figure S3). The FT-IR analyses of dry and wet sediments pointed on the presence of absorption bands at 2950 and 2850 cm^−1^ that are associated with –CH_3_ and –CH_2_– groups, confirming the presence of organic matter^20^ (Supplementary Figure S6). The FT-IR spectra of Amara sample show a detectable signal at 1725–1700 cm^−1^ that is characteristic for carbonyl groups in aldehydes, ketones and carbonic acids. The absorption bands in the spectral region of 1690–1500 cm^−1^can be associated with vibrations the carbonyl (C=O) and amino (–N–H) bonds of amides present in proteins, a finding that is corroborated by spectrophotometric protein quantification (Table 6). The aluminosilicates were detectable in all samples by the HO– specific broad FT-IR band between 3600 and 3650 cm^−1^ and absorption bands corresponding to Si–O–Si stretching (1040 and 860 cm^−1^) and Si–O–Si bending vibrational modes (465 cm^−1^) in addition to overtone of Al–O in Si cage (TO_4_) (1347–1360 cm^−1^) and Al–OH signals (891–936 cm^−1^). The doublet at 780–798 cm^−1^ is due to Si–O–Si inter tetrahedral bridging bonds in SiO_2_ and OH deformation band.

### Solid-state ^13^C- and ^1^H-NMR

The ^13^C ss-NMR spectra recorded by the CP-MAS technique (Supplementary Figure S7) indicate that the carbonyl/keto peak at 182 ppm, the carboxyl at 171.5 ppm, and the aliphatic peaks at 23.3 and 26.7 ppm, most probably associated to CH_3_/CH_2_ groups, are common to all three samples. Other aliphatic carbons peaking at 44.7 ppm were observed in Tekirghiol and Amara sapropels. The Tekirghiol sapropel is distinguished by the small peaks at 39.6 and 54.1 ppm assignable to a CH/CH_2_ group, the high intensity line at 33.7 ppm in which might be generated by molecules with many CH_2_ groups, for instance a fatty acid, and a low intensity NMR line, assignable to an aromatic carbon at 129.6 ppm. The ^1^H ss-NMR spectra (Supplementary Figure S7), are dominated by protons associated with the inorganic component of the sample (i.e., OH peak at − 3.4 ppm, the lines in the 4.7–5.2 ppm spectral range associated with water molecules in various binding environments). These lines can be correlated with the clay fraction identified in all the sediment samples by the mineralogical investigation. The ^1^H ss-NMR lines of the organic component are associated to the aliphatic signals in the 1–2 ppm range, the line at 8.9 ppm (hydrogen bonded carboxyl/amine), and possibly an aromatic proton at 5.8 ppm. The later can only be distinguished in the Tekirghiol sapropel, as there is no complete overlapping with the water NMR line.

### Isotope analysis and elemental analysis

Total organic carbon analysis indicated OC contents ranging from 2.09% (Tekirghiol) to 3.24% (Ursu). Total nitrogen (TN) concentrations are similar in Tekirghiol and Ursu sapropels (0.25 and 0.26%) and slightly higher (0.35%) in Amara sediment. C/N ratios (% w/w of combusted sediments) vary from 5.61% in Ursu, to 7.35% in Amara and 8.49% in Tekirghiol sample (Table 6). The values of *δ*^13^C_OC_ of organic matter in the carbonate-free sapropels were similar in Amara and Ursu lakes (− 27.77‰ and – 27.63‰, respectively) and slightly higher (− 23.87‰) in Tekirghiol Lake while δ^15^N values in bulk sapropels were 12.38, 5.13 and 4.98 inTekirghiol, Amara and Ursu lakes, respectively (Table 6). A large *δ*^34^S difference from sulfides to sulfates was identified in Tekirghiol and Ursu sediment pore waters (29.49‰ and 22.63‰, respectively), which is corroborated by soluble sulfides (HS– or H_2_S) detected in these sediments (Table 5).

### Pigment and lipid composition

Chlorophyll concentration was found to be highest in Ursu Lake (5490.86 µg L^–1^), compared to Tekirghiol Lake (3011.52 µg L^−1^) and Amara Lake (2348.8 µg L^−1^) (Table 6). Significant differences were detected between the fatty acids (FAs) profiles of the water samples as compared to sapropels, for each of the three investigated lakes (Supplementary Table 3). The prevalent saturated FAs were C16:0 (31.2–43.9%) and C18:0 (4.5–3.6%), along with the abundant unsaturated FAs C16:1 n-9 (1.4–23.4%, highest in Ursu Lake surface), C18:1 n-9 (7–23.5%, highest in Tekirghiol water), C18:2 n-6 (3.0–10.5%, highest in Amara water and sapropel), respectively C18:3 n-3 (1.4–24.2%, highest in Amara water). The monounsaturated C16:1v7 isomer, characteristic of bacterial sources was abundant in Ursu sapropel.

## Discussion

**Figure 7:**
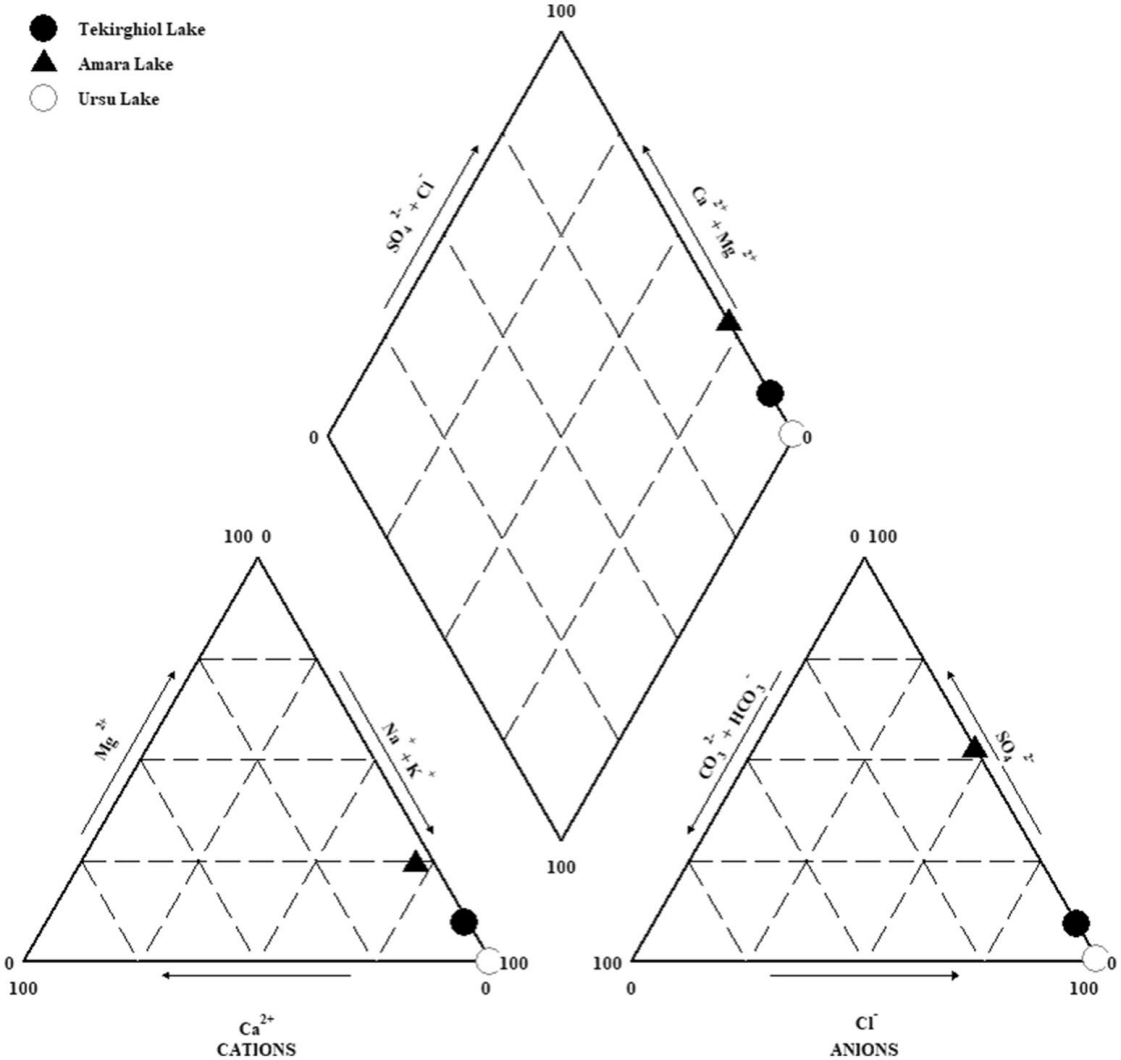
Piper diagram-based hydrochemical classification of Tekirghiol (full circle), Amara (full triangle) and Ursu (open circle) lakes waters.

Based on the chemical analyses of sapropels and brine water, Tekirghiol and Ursu lakes can be classified as Na–chloride type and Amara Lake water as mixed Na–sulfate–chloride type (Fig. 7). The water collected in the northern littoral area of Tekirghiol Lake is oxygenated and saline (> 10% estimated salinity) with ionic composition resembling that of sea water thus allowing us to consider this lake as thalassohaline^21^ (Supplementary Table 1). Amara Lake’s water is slightly basic (pH ∼ 8.8), oxygenated and brackish (∼ 1% total salinity), with Na^+^, Mg^2+^, SO_4_^2−^ and Cl^−^ as major cations and anions respectively, indicating its athalassohaline, continental origin. The high sulfate content gives the specific bitter (Rom., “amar”) taste and thus the name of the lake. The slightly basic and oxygenated waters of Tekirghiol and Amara lakes could be explained by their shallowness and good water mixing that in combination with light transparency allow development of phytoplankton and subsequent pH increase due to CO_2_ removal by photosynthetic activity^22^. The water atop deep sapropel in Ursu Lake was slightly acidic (pH ∼ 6), hypersaline (∼ 40% total salinity) and euxinic (O_2_-depleted, sulfide-enriched and highly reduced). The bottom water of Ursu Lake was shown to harbor heterotrophic fermentative microbial community that might be responsible for CO_2_ production driving the slight decrease of pH^23,24^. Within the sapropels, most of the salinity-contributing minerals (mainly Na and Cl) were found under soluble form (i.e., in pore water), whereas K, Ca, Mg, Fe, and Mn were under solid form, pinpointing that the chemical composition of sapropels (Tables 2, 3) is strongly related to their mineralogical fraction. Concerning the purity requirements of peloids used in balneotherapy, related to the toxicity and possible resorption through the skin of bioavailable elements, the values obtained for all three sapropels are within the range (or lower) detected for therapeutic muds in Croatia, Spain or Slovenia^12,25,26^, and thus precluding the risks of metal toxicity.

Regarding the higher concentration of strontium detected in Tekirghiol sapropel, compared to the other two sapropels, the element is generally found in significant amounts in marine evaporites, and due to the similarity of ionic radius, Sr could be incorporated into calcite or aragonite, by substituting the Ca in these minerals^25,27^. Carretero et al.^26^ noted that microelements show limited or no mobility, thus having little effect on the quality of sapropels used in pelotherapy. Lanthanide composition of Tekirghiol and Amara sediments are comparable with those reported in the Darscho and Neusiedlersee salt lakes sediments in Austria^28^ (Supplementary Table S2). The shared characteristics of these lakes are the higher concentrations of light compared to that of the heavy rare elements as well as the lanthanides that have an even atomic number compared to those with odd atomic number. The concentrations of rare elements in the sediments of Ursu Lake are comparable to those in the sedimentary rocks of acidic Colour Lake located in the Arctic region of Canada^29^.

**Figure 8:**
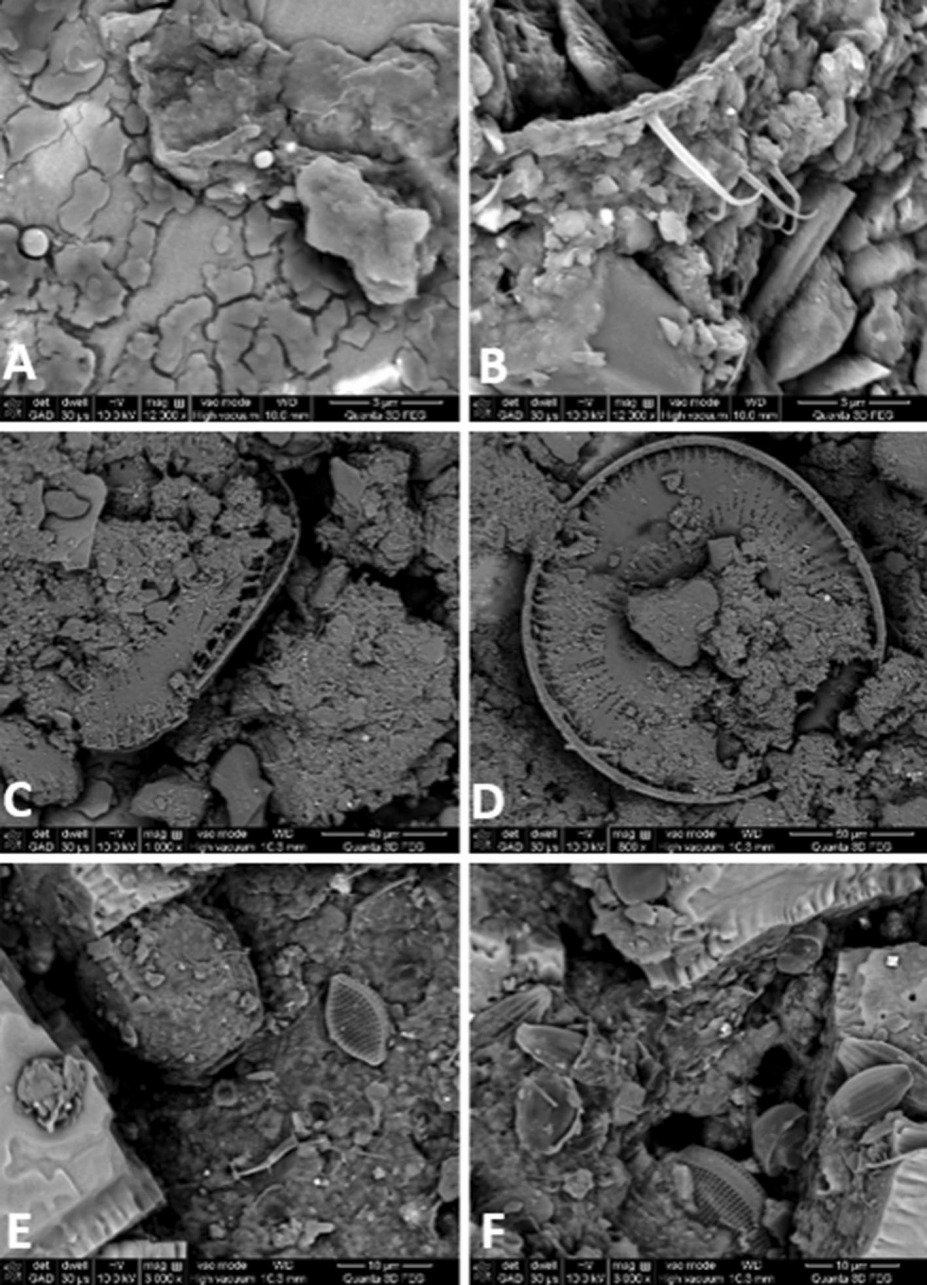
Scanning electron microscopy (SEM) photographs of sediment samples from Tekirghiol (**A**, **B**, 12,000× magnification), Amara (**C**, **D**, 1000×) and Ursu (**E**, **F**, 3000×) lakes, revealing details of phyto- and zooplankton and inorganic morphology.

The significant phyto- and zooplankton debris found in Ursu Lake sapropel (Fig. 8) could be important contributors to the organic matter in these sediments. Chlorophyll concentration, used as a proxy for microphytobenthos abundance is highest in Ursu Lake and may derive from algal necromass and/or inactive biomass that has drifted downwards from the oxygenated upper strata^24^. Fatty acids (FAs) inventories of water column and sapropels suggest the substantial contribution of algae and cyanobacteria to the settling organic matter^30^, as the ubiquitous planktonic and bacterial lipids (C16:0 and C18:0) dominated the profiles. The high occurrence of polyunsaturated FA C18:1 n-9 fatty acids can reflect several different sources, i.e., green algae, bacteria, zooplankton, or higher plants, while C18:2 n-6 known as markers of macroalgae^31^. The high abundance of cyanobacteria and diatoms observed in the tested lakes may further provide organic compounds with beneficial effects on the maturation processes and therapeutic properties of sediments^6^.

The *δ*^13^C_OC_ and δ^15^N values in sediments were closely similar to those measured in particulate matter retained from overlying water (Supplementary Table S1) thus possibly indicating that the most of buried organic carbon and nitrogen derives from the settling of organic matter in the water mass. *δ*^13^C values in Amara and Ursu are typical for lacustrine algae and the C/N ratios < 10 for all three lakes point to the autochthonous origin of the organic matter^32^. All investigated lakes have been long-time exploited for touristic and medical purposes and, in addition, Amara and Tekirghiol lakes have also experienced intense agricultural input from the vicinity. The *δ* ^34^S of sulfates in pore water of Amara sediments is similar to that of seawater sulfates (+ 20‰) and to the sulfate-enriched uppermost layers of marine sediments in contrast to Tekirghiol and Ursu sediments that are enriched in heavier ^34^S (both for sulfates and bulk sulfur) and follow the trend in decreasing sulfate contents^33^. The increased heavy ^34^S isotope in sulfides from Amara sediment pore water may suggest its preferential binding as metal sulfides (pyrite) in agreement with our elemental mapping and FT-IR findings. The differences in *δ*^34^S of sulfides and sulfates in Tekirghiol and Ursu sediment pore waters corroborated by the presence of soluble sulfides (HS– or H_2_S) in these sediments provide interesting evidence towards the prevalence of microbial sulfate reduction^34^. Soluble sulfides were shown to source for ^34^S-enriched organosulfur compounds in anoxic sediments^35^. Although limited analytical data are preventing us to further connect the stable isotope fractionation data and organic matter origin, we could fairly suggest that the higher bulk *δ* ^34^S in Tekirghol and Ursu sediments as compared to Amara may indicate timely formation of organosulfur compounds in these sediments. Further insights into the organic matter composition and origin, provided by ssNMR, FT-IR and Raman spectroscopy indicate specific signatures associated to carotenoids (Ursu) and β-carotene (Tekirghiol and Amara), proteins and kerogen. Kerogen is a mixture of insoluble, solid hydrocarbon-based organic compounds occurring in sedimentary source rocks and its remarkable presence in Tekirghiol sapropels would suggest an initial stage of sediment maturation^36^.

The physical and physico-chemical properties of the three sapropels are tightly bound to their mineralogical composition and textural disparities. The higher clay concentration and predominance of smectite and interstratified illite/smectite R0 (60/40) in Ursu sapropel favored increased water content (∼ 70%). Water retention was reduced in Amara sapropel, which displayed similar clay fraction composition, however, the clay content was lowest of all three sapropels, and the sand fraction was > 35%. The majority of studies on peloids used for therapy report on a mixed composition of the solid phase, which includes clay (e.g., smectite, montmorillonite, kaolinite) and non-clay minerals (quartz, calcite, feldspars, etc.), in various proportions^6,12,25,37,38^. The mineral composition of sapropels controls the physical and chemical properties and thus the suitability of sediments to be used for therapeutic purposes, an increased clay content assuring better physico-chemical properties of muds^12,15^. Due to the relatively high smectite content, all three sapropels can be considered suitable for pelotherapy, as smectite materials have the capacity to retain large water quantities, present high exchange and high-heat capacity^6^. The presence of carbonates within the sapropels of Tekirghiol and Amara might be beneficial for therapy, as it was found that carbonates stimulate subcutaneous circulation and optimal stratification of the epidermis^15^.

Increased silt fraction in Ursu and Tekirghiol sapropels, compared to Amara are related to higher NaCl concentrations, which might influence the aggregation state of clay^38^. Increased silt:clay ratio and organic matter content, i.e., pigments of diatoms and cyanobacteria, protein signatures from microorganisms, also detected by SEM and FT-IR, could account for the reduced BET specific surface area of Amara and Ursu sapropels. SSA is a factor to be considered when addressing the therapeutic effects of muds, peloids or sapropels, and is correlated to clay minerals composition and grain size. SSA assumes different values for various clays, ranging from 1 to > 100 m^2^ g^−1^, depending on the mineral structure^39^. Resistant minerals (i.e., quartz) contribute mainly to the coarse fraction while aluminosilicates (i.e., smectite, illite, kaolinite, chlorite) determine the fine-grained nature of sapropels. The sapropels from Tekirghiol, Amara and Ursu lakes are enriched in quartz and calcite these minerals showing in general two, or even three orders of magnitude lower SSA compared with some clay minerals^40^. The SSA of these sapropels are within the range recorded for peloid mud from Morinje Bay (eastern Adriatic coast, Croatia)^12^ or black mud from Greece or the Dead Sea^41^. Depending on the composition, structure, and environmental conditions such as salinity, temperature, pH and hydrodynamic conditions, organic matter can lower the SSA due to the adsorption of organic material to clay minerals surfaces, thus reducing the availability of external surfaces^38^.

The presence of organic matter and the type of clay material could control the CEC of the sapropels. The total cation exchange capacity of the three sapropels was in the range of other muds used for therapy in Spanish spas (11–112 cmol(+)/kg)^6^, or Turkey (9.2–32.2 cmol(+)/kg)^2^. The higher CEC values in Ursu Lake could be equivalent to higher absorption, and thus better therapeutic effects, as the sapropel may sequester an increased amount of trace elements, essential for the therapeutic potential^2^. CEC and thermal properties are related to the therapeutic effect of peloids, as peloidotherapy is in most cases associated with thermotherapy^7^. Compared to Tekirgiol and Amara sapropels, Ursu sapropel showed the greatest heat capacity and thermal retentivity, and lower thermal conductivity and diffusivity coefficients (Table 3). These properties ensure the maintenance of a high temperature of therapeutic muds during application time, and are positively influenced by higher water content of the sapropel^37^. The thermal diffusivity determined for Tekirghiol and Amara sapropels are higher than the values obtained for the DAX peloid (TERDAX®)^37^, and in the range of the saline peloids analysed by Glavaš et al.^25^, while Ursu sapropel shows the lowest thermal diffusivity coefficients of all peloids characterized^7^. Thermal conductivity measurements for Tekirghiol and Amara sediments were similar to TERDAX® and Spanish clays but lower than Slovenian sandy sediments. In terms of thermal conductivity and retentivity, Ursu sapropel show similar thermal characteristics to TERDAX®, Spanish clays and silty sediments from Sečovlje peloids^25,37,38^. Specific heat measurements are within the range of those determined for bentonite clays from Italy^1^, or sandy silt sediments from Croatia or Slovenia^12,25^.

## Conclusions

The current study reports the mineralogy, physical properties and chemistry of organic carbon-rich sediments from three Romanian lakes with contrasting salinities. The sapropel samples collected from the three lakes consist mainly of quartz, calcite, aragonite, and halite, the latter found in high concentration in the hypersaline Ursu sapropel. The clay composition of the fraction < 2 μm indicates similar clay minerals in all three sapropels, with higher proportions of illite in Tekirghiol, and higher smectite and interstratified Illite/Smectite R0 (60/40) content in Ursu and Amara sapropels. The presence of smectite and the increased mud fraction as well as low concentrations of potentially toxic microelements, support the therapeutic potential of all three sapropels. Particle size analysis, physical properties and thermal behavior point out that Ursu and Tekirghiol sapropels are better suited for pelotherapy treatments.

## Materials and methods

### Site description and sampling

**Tekirghiol Lake** (44° 03′ N, 28° 36′ E; 0.8 m a.s.l.; 11.6 km^2^ area; maximum and average depths of ∼ 9 m and ∼ 3 m, respectively) is located in the south-eastern part of Romania (Fig. 1). Considered the largest Romanian saline lake^42^, Tekirghiol is a shallow, saline coastal lake separated from the sea by a 200 m sand barrier. Its salinity and chemical composition result from intense evapo-concentration due to the local climate aridity and low freshwater input^22,42^. Tekirghiol Lake is a natural bird reserve and has been exploited for recreational and therapeutic purposes since the mid-nineteenth century^43^. **Amara Lake** (44° 36′ N, 27° 20′ E; 32 m a.s.l.; 1.3 km^2^ area; maximum and average depths of ∼ 6 m and ∼ 2 m respectively) is a brackish oxbow lake located in the eastern part of the Romanian Plain^44^. In the last century, the lake witnessed an accentuated desalinization, from ∼ 90 gL^−1^ in 1987 to 4.5 gL^−1^ in 1973^44^, while more recent results indicate salinity values of 9.43 gL^−1^^45^. Amara Lake is the largest chloride-sulfated plain lake in Romania and has a protected status as bird natural reserve and its water and muds have long been utilized for bathing and medicinal purposes. **Ursu Lake** (46° 36′ N, 25° 05′ E; 505 m a.s.l.; 4.1 km^2^ area; maximum and average depths of ∼ 18 and ∼ 12 m, respectively), located in the Transylvanian Basin (Central Romania), is considered one of the largest heliothermal, freshwater-to-hypersaline meromictic lake in the world^14,24^. Ursu Lake has formed by intense salt dissolution of underlying rock salt between 1875 and 1880; it is currently sustainably exploited for therapeutic and recreational purposes while having a status of natural hydrogeological reserve^14^.

Water and sediment sampling were performed in October 2017 from areas where muds are extracted for pelotherapy purposes. Water was collected in 1 L sterile flasks by 12 V submersible pump from 0.5, 2 and 12 m-water depths from Tekirghiol, Amara and Ursu lakes, respectively. In-situ physico-chemical variables of water (temperature, pH, dissolved oxygen, oxido-reduction potential, electrical conductivity and total dissolved solids) were measured by portable multiparameter model HI 9828 (Hanna Instruments, USA) as described in^24^. Sediment sampling was performed as described in^18^, at water depths of 1 m, 2 m and 13 m in Tekirghiol, Amara and Ursu lakes, respectively. Briefly, mixed sediment samples (obtained from three casts, within 2 m radius) were collected using a custom sediment corer (made of pieces of 1 m length each, 10 cm inner diameter, 78.5 cm^2^ collecting area) from Tekirghiol Lake, and a Petite Ponar grab from Amara and Ursu Lakes.. Sediment samples were homogenized, stored at 4 °C in sterile 1-L containers and processed within 24 h.

### Mineralogical, differential thermal (DTA) and thermogravimetric (TGA) analyses

Quantitative mineralogical analysis of sapropels collected from Tekirghiol, Amara and Ursu lakes was performed by a commercial analytical company (QMineral BV, Belgium) following standardized methods. For determination of the bulk mineralogical composition, powdered samples were ground in a wet milling device in ethanol. After drying, the samples were treated in a way to avoid preferred orientation, loaded in XRD sample holders, and measured by X-ray diffraction using CuKα radiation. The subsequent identification was performed by comparison of the positions and intensities of the reflections with those of the minerals in the available public databases (COD). The quantification was performed by an in-house method based on the Rietveld method. For the clay mineralogical analysis (fraction < 2 μm), 3 g of each sample was selectively separated by centrifugation after a thorough chemical treatment to remove cementing agents, as carbonates, Fe-oxides and hydroxides, and organic matter. Subsequently, the exchangeable cations were exchanged to their Ca^2+^-form. Preparations yielding highly oriented clay particles were obtained by sedimentation and were subsequently analysed by X-ray diffraction. The detailed clay mineralogical composition of the < 2 μm fraction was obtained by modeling using Newmod II software (https://newmod-for-clays.com/)^46^. For DTA and TGA analyses, samples were dried at 40 °C, placed in an alumina crucible and measured with a Netzsch STA 409 PC from room temperature to 1000 °C in an inert N_2_-atmosphere. The precision of XRD measurements in mainly determined by preparation of samples. For the three sapropel samples, we optimized and tested so that for three consecutive measurements there is virtually no difference in the three diffraction patterns. The accuracy on the mineral quantities depends on the sample matrix and the largest error derives from user interpretation. The accuracy for non-clay phases was around 0.5%, and for the clay phases ca. 2–3%.

### Physical properties

The grain-size distribution of the samples was measured by laser diffraction using the Coulter LS13-320 Aqeuous Liquid Module instrument, after wet dispersion. For cation exchange capacity (CEC), the samples were first washed with milli-Q water and centrifuged to remove soluble salts. A representative part of each sample was first dried at 110 °C, and total CEC was determined by Co (III)-hexamine method, as described by Bardon et al.^47^.

The specific surface area (SSA) measurements were performed before and after desalinization of sapropels by washing with ultrapure water at 1:3 sample/water volume ratio. The results were calculated from N_2_-adsorption–desorption isotherms using a QSurf M1 Surface Area analyzer (Thermo Fisher Scientific, USA). The SSA was obtained by single point Brunauer–Emmett–Teller (BET) method, while the total pore volumes were determined by the Barret–Joyner–Halenda (BJH) method^48^. Density measurements were performed using a pycnometer on rehydrated sediment samples (by mixing sediment to water at 32% to 68%, respectively) according to Glavaš et al.^25^. Thermal characterization was performed in triplicate, using photopyroelectric (PPE) calorimetry, as described in^49^. Thermal diffusivity was measured directly, by the back detection PPE configuration with opaque sample and thermally thick sample and sensor. The scanning procedure was the so-called thermal-wave resonator cavity (TWRC) method and was performed for sample’s thickness. Thermal effusivity was measured in front PPE detection configuration with thermally thin and optically opaque sensor. As the effusivity can be expressed as the square root of the product of thermal conductivity and specific heat, and thermal conductivity can be expressed as the product of thermal diffusivity and volume specific heat^50,51^ we were able to calculate the specific heat and thermal conductivity of the sediment samples. Thermal retentivity was calculated as mentioned in^5^.

### Chemical analyses

For the chemical analyses, three technical replicates of the homogenized samples prepared as described in *Site description and sampling* were used. The leachable major ions were water-extracted using sediment-to-ultrapure water ratio of 1:10 at room temperature. The suspension was centrifuged and the supernatant was filtered through 0.22 μm-pore sized PTFE membranes. The obtained filtrate was further analyzed for ion content. Na^+^, K^+^, Ca^2+^, Mg^2+^, total P, Fe, and Mn, as well as the rare earth elements (lanthanides) were measured by inductively coupled plasma atomic emission spectrometry (ICP-AES) using Optima 5300 DV spectrometer (Perkin Elmer, USA). Chloride (Cl^−^) was measured by titrimetric method. Sulfate (SO_4_^2−^) was assessed by ion chromatography on ICS-1500 (Dionex, USA). Dissolved total carbon (DTC) and dissolved inorganic carbon (DIC) were measured by catalytic combustion and infrared detection of CO_2_ using a Multi N/C 2100S Analyser (Analytik Jena, Germany). Dissolved organic carbon (DOC) was obtained by subtracting DIC from DTC. Total dissolved nitrogen (DTN) as bound nitrogen (including free ammonia, ammonium, nitrite, nitrate, and organic nitrogen) was analyzed by catalytic combustion followed by oxidation of NO to N_2_O with ozone and subsequent chemiluminescence detection. Ammonium, nitrate and nitrite ions were analyzed by Lambda 25 UV–VIS spectrophotometer (Perkin Elmer, Beaconsfield, UK) following formation of colored complexes: indophenol blue complex (ammonium), yellow complex formed with sulphosalicylic acid (nitrate), and red colored azo dye formed from diazonium salt in the presence of N-(1-Naphthyl) ethylenediamine and sulphanilamide under acidic conditions (nitrite). The concentration of sulfides was determined by methylene blue method after fixation of samples with 2% (v/v) Zn-acetate. Humidity was estimated by loss-on-ignition (LOI) method following oven-drying of sediments at 105 °C for 24 h. The pH and salinity of pore water were measured with a portable HI 9828 multiparameter (Hanna Instruments, USA). Methane concentration in water was determined as described in^23^. Protein concentration from 2 M NaOH alkaline extract of wet sediments (1:1, v/v) was determined by Lowry methods using bovine serum albumin as standard. PCoA analysis was performed in R software v4.0.4 (https://www.r-project.org/)^52^. Piper diagram was created by using GW Chart v1.30^53^.

### Raman spectroscopy

FT-Raman spectra of the bulk samples employed an Equinox 55 FT-IR spectrometer (Bruker Optik GmbH, Germany) with an integrated FRA 106S Raman module. Spectra (4 cm^−1^ resolution) were measured following excitation by Nd:YAG laser operating at 1064 nm and detection by Ge detector operating at liquid nitrogen temperature. Due to high fluorescence of samples, low laser power between 85 and 100 mW was applied, to achieve a reasonable signal to background ratio. Confocal micro-Raman spectra were acquired by InVia reflex Raman system (Renishaw, UK), by excitation with the conventional 532-nm line from a Cobolt diode pumped solid state laser using very short exposure due to the considerable intrinsic fluorescence of the mud. Typical acquisition parameters comprised 1 s, 1 acquisition and 1–2 mW. Single spectra have been collected from random spots observed through the microscope equipped with 20× and 100× objectives, in the 50–1836 cm^−1^ range, with a spectral resolution of 0.5 cm^−1^. Streamline imaging has been applied on small rectangle areas of the video images to assess the photosynthetic pigment-bearing microorganisms associated with the presence of carotenoid resonance Raman signal selectively detected under 532 nm excitation^54^. To further decrease the interference of high intrinsic fluorescence, a SERS experiment was run by coating the mud-containing slides with citrate-reduced silver nanoparticles (AgNPs) prepared according to Lee and Meisel^55^. AgNPs-coated sapropel films were tested for the signal-to-background resonance Raman signal combined with SERS effect^56^.

### Isotope and elemental analysis

Analysis of δ^13^C, δ ^15^ N, δ ^18^O and δ ^34^S in bulk sapropels and water was performed by standardized Elemental Analyser-Isotope Ratio Mass Spectrometry (EA-IRMS) at IsoAnalytical Co. Ltd. (UK). The organic carbon δ ^13^C was measured in acidified, carbonate-free sediment and using IA-R001, IA-R005 and IA-R006 standards. Following the water filtration through 0.22 μm pore sized membranes, the δ^13^C and δ^15^N were analyzed from retained particulate matter by EA-IRMS. During δ^15^N analysis, the IA-R001, IA-R045 and IA-R063 standards were used. δ^34^S of sulfides and sulfates in the sediment pore water and overlying water mass were analyzed from the zinc-acetate and BaCl_2_ precipitates, respectively and using IA-R061, IA-R025, and IA-R026 as in-house standards. δ^13^C and δ^18^O in dissolved inorganic carbon (DIC) or sediment carbonates were measured by acidification of sample followed by CO_2_ release and subsequent CF-IRMS (Europa Scientific 20-20, PDZ Europa, Crewe, UK) and using IA-R022, NBS-18, IA-R066 and IAEA-603 standards. Elemental analysis of C and N and subsequent C/N ratio from bulk sediments were performed by sample combustion at 1700 °C using Europa Scientific elemental analyzer. Total organic carbon (TOC) content was analyzed by QMineral BV (Belgium). Briefly, sediment samples were weighed, mixed with 10% HCl solution and heated at 60 °C for several hours to dissolve any inorganic carbon. After the dissolution reaction, the concentrates were transferred to a Carlo Erba EA1108 elemental analyzer where the samples are first fused at 1600–1800 °C and the TOC content measured by chromatography.

## Supplementary Information

Supplementary Information.
