# Supplementary material for: Comprehensive mineralogical and physicochemical characterization of recent sapropels from Romanian saline lakes for potential use in pelotherapy
Source: Sci Rep. 2021 Sep 20;11:18633. doi: 10.1038/s41598-021-97904-1 (PMC8452724; doi:10.1038/s41598-021-97904-1)
Supplement: Supplementary file 1 — Supplementary Information. [file 41598_2021_97904_MOESM1_ESM.pdf]

Supplementary Materials

Comprehensive mineralogical and physicochemical characterization of recent sapropels from Romanian saline lakes for potential use in pelotherapy

Andreea Baricz[1*], Erika A. Levei[2], Marin Șenilă[2], Simona Cîntă Pînzaru[3], Mihaela Aluaş[3], Adriana Vulpoi[4], Claudiu Filip[5], Carmen Tripon[5], Dorin Dădârlat[5], Doriana M. Buda[1,6], Francisc V. Dulf[7], Adela Pintea[8], Adorján Cristea[1,6], Vasile Muntean[1], Zsolt G. Keresztes[1], Mircea Alexe[9] and Horia Leonard Banciu[1,6 *]

1 - Department of Molecular Biology and Biotechnology, Faculty of Biology and Geology, Babeş-Bolyai University, 5-7 Clinicilor Str., 400006 Cluj-Napoca, Romania

2 - INCDO-INOE 2000, Research Institute for Analytical Instrumentation, 67 Donath Str., 400293 Cluj-Napoca, Romania

3 - Faculty of Physics, Babeş-Bolyai University, 1 M. Kogalniceanu Str., 400084 Cluj-Napoca, Romania

4 - Institute for Interdisciplinary Research in Bio-Nano-Sciences, Babeş-Bolyai University, Cluj-Napoca, Romania

5 - National Institute for Research and Development of Isotopic and Molecular Technologies, 67-103 Donath Str., 400293 Cluj-Napoca, Romania

6 - Centre for Systems Biology, Biodiversity and Bioresources, Babeş-Bolyai University, 400006 Cluj-Napoca, Romania

7 – Department of Engineering and Environmental Protection, University of Agricultural Sciences and Veterinary Medicine Cluj-Napoca, 400372 Cluj-Napoca, Romania

8 - Department of Chemistry and Biochemistry, University of Agricultural Sciences and Veterinary Medicine Cluj-Napoca, 400372 Cluj-Napoca, Romania

9 - Department of Physical and Technical Geography, Faculty of Geography, Babeș-Bolyai University, 5-7 Clinicilor Str., 400006 Cluj-Napoca, Romania

*Corresponding authors:
Andreea Baricz, e-mail: andreea.miclea@ubbcluj.ro and
Horia Leonard Banciu, e-mail: horia.banciu@ubbcluj.ro

Running Title: Comprehensive characterization-of recent saline sapropels

Table S1. *In situ* measurements of physico-chemical parameters and water chemistry (average±standard deviation of three replicates) for Tekirghiol, Amara and Ursu lakes during October 2017.

**Physico-chemical parameter/ Compound**	**Tekirghiol Lake**	**Amara Lake**	**Ursu Lake**
Depth (m)	0.5	2	12
Temperature (^oC)	15	15.79	21.6
pH	8.39	8.77	6.13
Dissolved oxygen, DO (mg L^{-1})	5.69	9.79	0.48
Redox potential, ORP (mV)	-48.1	189.6	-358.5
Electrical conductivity, EC (mS/cm)	105.7	18.3	350
Total dissolved solids, TDS (g L^{-1})	52.8	9.15	380
Na^+ (mg L^{-1})	41500	2270	180000
K^+ (mg L^{-1})	1160	83	134
Ca^{2+}(mg L^{-1})	282	110	505
Mg^{2+} (mg L^{-1})	2360	413	9.1
Cl^- (mg L^{-1})	58500	3550	245000
Total Fe (mg L^{-1})	<0.05	<0.05	<0.05
Total Mn (mg L^{-1})	<0.01	<0.01	<0.01
SO_4^{2-} (mg L^{-1})	8060	5325	2445
Estimated salinity as sum of ions (g L^{-1})	111.82	11.75	428.09
Total sulfides (S^{2-}) (mg L^{-1})	<0.02	<0.02	94.8
TP (mg L^{-1})	<0.05	<0.05	0.52

TN (mg L^{-1})	6.2±0.3	7.6±0.4	37±2
DTN (mg L^{-1})	4.8±0.2	5.5±0.3	34±2
NH_4-N (mg L^{-1})	1.75±0.10	1.36±0.08	35.95±1.70
ON (mg L^{-1})	4.45±0.20	6.24±0.28	1.05±0.05
TC (mg L^{-1})	144±6	80±5	189±8
TOC (mg L^{-1})	66±3	16±1	36±2
DC (mg L^{-1})	138±7	76±4	93±5
DOC (mg L^{-1})	64±3	14±1	20±1
$\delta^{13}C$ (‰) [a]	-23.75	-29.07	-28.41
$\delta^{13}C_{DIC}$ (‰) [b]	-2.08	-4.16	-17.48
$\delta^{15}N$ (‰) [a]	11.83	7.72	6.51
$\delta^{34}S$ sulfides (‰) [c]	Nm	Nm	-6.88
$\delta^{34}S$ sulfates (‰) [c]	18.86	8.11	17.69
$\delta^{18}O$ (‰) [d]	-1.58	0.24	-9.67
C1-hydrocarbon (CH_4) (mg L^{-1})	0.0205	0.0034	0.1017

[a] from particulate matter retained onto 0.22 µm pore-sized membrane following filtration of lake water; [b] – following water acidification; [c]- from lake water treated with Zn-acetate (for sulfides recovery) or $BaCl_2$ (for sulfates recovery); [d] – from lake water distilled by azeotropic distillation prior to analysis; Nm –not measured.

Table S2. Comparative contents of rare earth elements (lanthanides) in sediments of tested lakes and Darscho and Neusiedlersee lakes (Austria) [1], and Colour Lake (Canada) [2], respectively.

Rare earth elements (mg/kg)	**Tekirghiol Lake**	**Amara Lake**	**Ursu Lake**	**Darscho Lake**	**Neusiedlersee Lake**	**Colour Lake**
La	10.33	8.57	1.99	10.2	9.10	1.87
Ce	22.56	20.17	5.19	20.5	17.0	4.21
Pr	2.82	2.51	0.69	Nd	Nd	0.55
Nd	11.25	10.22	2.69	8.29	6.76	2.20
Sm	2.58	2.47	0.67	1.84	1.31	0.38
Eu	0.63	0.65	0.20	0.41	0.31	0.08
Gd	2.88	2.76	0.78	1.66	1.22	0.33
Tb	0.41	0.40	<0.33	0.26	0.17	0.05
Dy	1.86	1.81	0.54	Nd	Nd	0.29
Ho	0.83	0.37	0.15	Nd	Nd	0.06
Er	0.96	0.92	<0.33	Nd	Nd	0.17
Tm	<0,33	<0.33	<0.33	0.26	0.25	0.02
Yb	0.73	0.69	0.26	0.86	0.54	0.15
Lu	<0.33	<0.33	<0.33	Nd	Nd	0.02
Total	57.84	51.54	13.16	44.28	36.66	10.38

Note: Detection limit was 0.33 mg kg^{-1} sediment; Nd –not determined.

Table S3. GC-MS analysis of bacterial acid methyl esters extracted from water and sediments of three Romanian salt lakes.

Peak No.	Fatty acid	% from total fatty acids						
		Tekirghiol Lake		Amara Lake		Ursu Lake		
		Water	Sed.	Water	Sed.	Water (3 m)	Water (11 m)	Sed.
1	(C8:0)	0.32	1.22	0	1.30	0	0	0.45
2	(C10:0)	0	0.66	0	1.15	0	0	0.28
3	(C12:0)	0	0.97	0	1.08	0.92	0.59	0.49
4	(C14:0)	2.34	3.58	3.46	5.56	4.93	4.34	5.74
5	(C15:0)	0	0.42	2.04	0.35	2.96	1.10	1.46
6	12-methyltetradecanoate (a C15:0)	0	0.38	0	0.47	0.76	0	0.30
7	14-methylpentadecanoate (i C16:0)	0	1.29	0	1.58	0.24	0.80	0.68
8	(C16:0)	37.52	34.20	31.29	33.34	43.99	38.82	34.47
9	(C16:1 n-9)	1.70	4.78	1.45	7.11	23.43	4.00	1.66
10	(C16:1 n-7)	2.21	3.10	4.80	2.44	2.57	8.16	9.37
11	(C18:0)	9.64	12.69	8.69	13.62	4.54	11.61	9.68
12	(C18:1 n-9)	23.50	17.60	7.07	14.84	5.95	18.50	13.58

13	(trans-C18:1 n-9)	7.83	1.27	6.42	3.45	5.21	4.33	1.44
14	(C18:2 n-6)	4.67	12.23	10.59	10.47	3.03	4.98	8.40
14'	(C18:3 n-3)	10.28	1.54	24.21	0.92	1.47	1.05	5.31
15	(C20:0)	0	1.52	0	1.77	0	0.45	1.78
16	2-OH C16:0	0	2.56	0	0.55	0	1.28	4.90

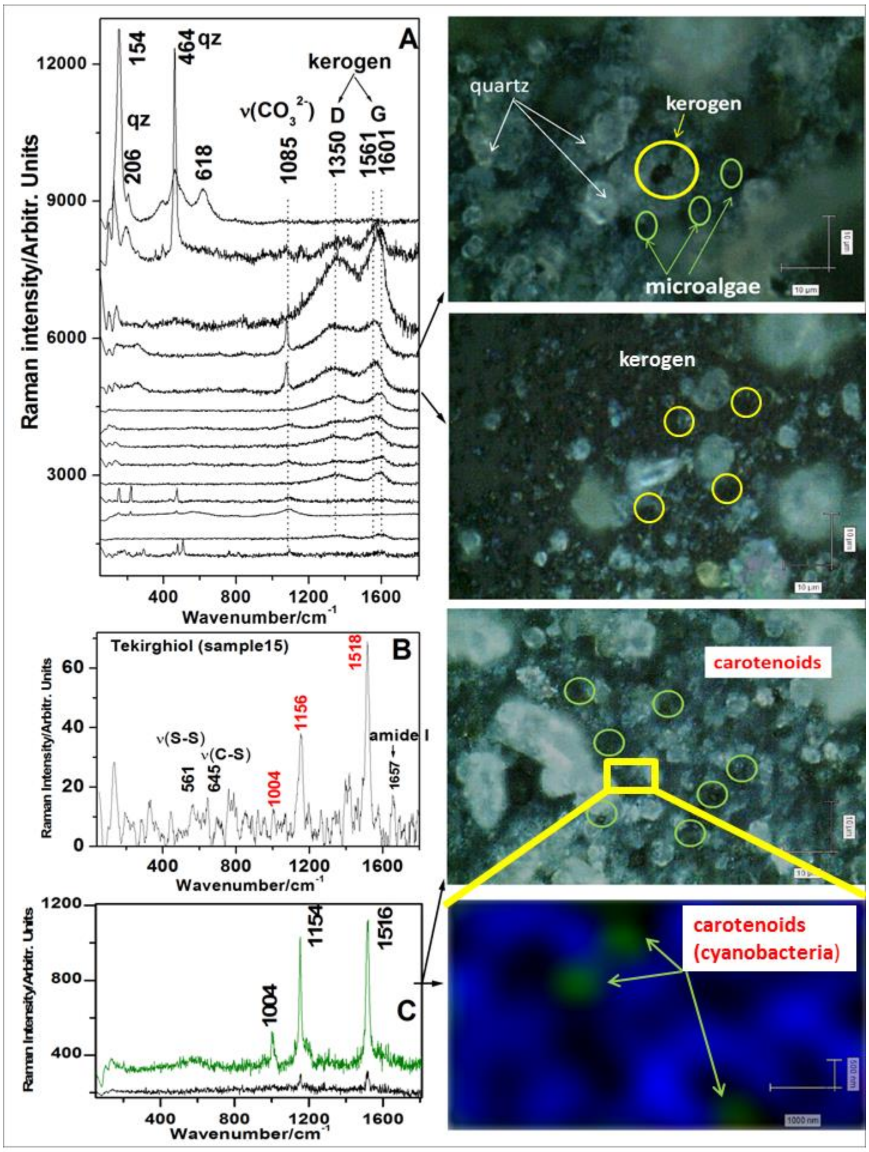

Figure S1. **Raman spectroscopy briefing the dominant Tekirghiol sapropel composition**. Series of spectra featuring kerogen A) along quartz and carbonate mineral; typical carotenoids signal accompanied by organic trace of proteins B); and typical, clear RR spectra of carotenoids C); Corresponding light microscopy images taken with Raman microscope during measurements, highlight the inorganic bright crystals, black spots of kerogen and dark green microorganisms signaling carotenoids. A small map (bottom, right) of 5 µm x 3 µm illustrates quartz signal distribution (blue) embedding green spots (< 1 µm) of carotenoid signal, most probably from cyanobacteria. Excitation: 532 nm.

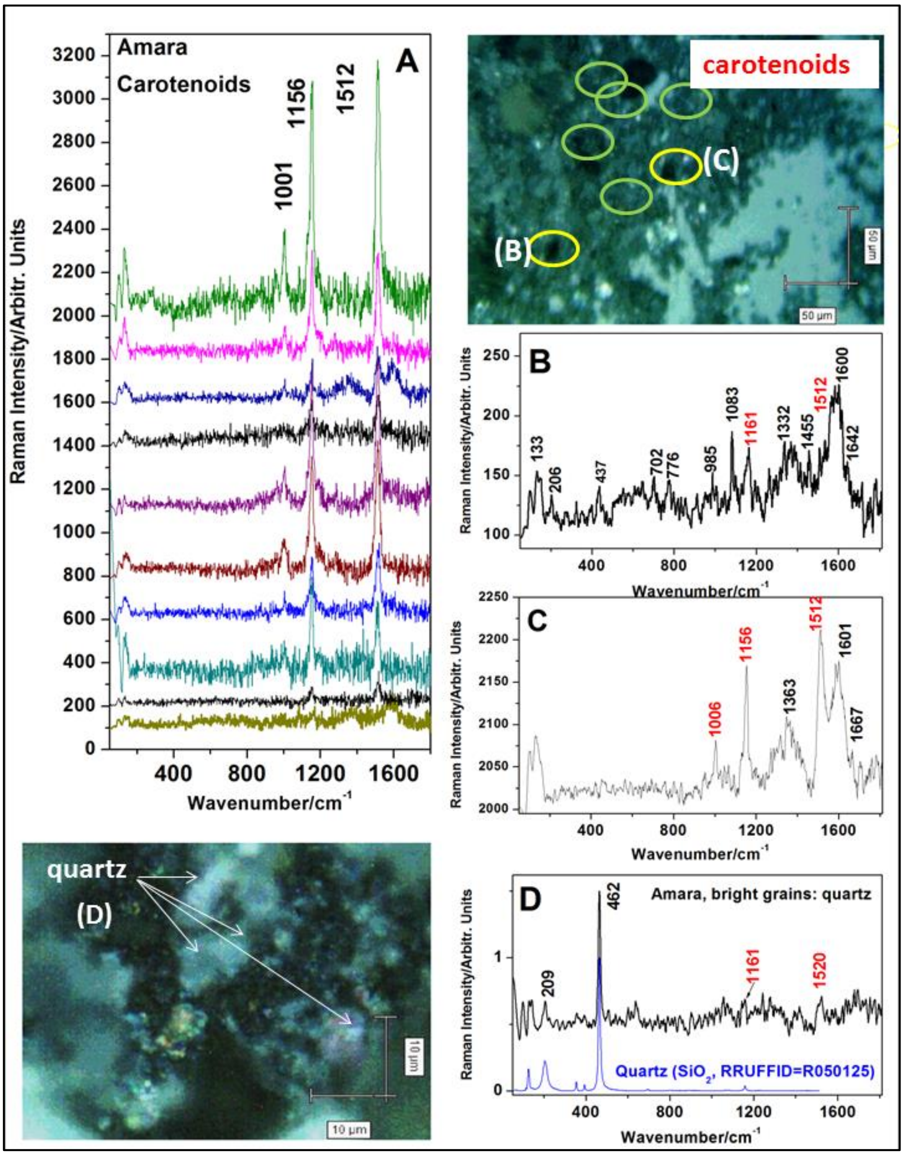

Figure S2. Typical micro-Raman spectra collected from raw mud from Amara. Series of background subtracted spectra showing mainly carotenoid bands (A) at 1512 (C=C mode) and 1156 cm^{-1} (C-C) mode and trace of organic (B, C) or inorganic (D) matter. The carotenoid bands are plotted in red (B, C, D), while D shows a typical signal of quartz. Micrographs collected from the single point spectra taken in B, C, D are showed in the right. Bright grains are associated with quartz crystals while dark, spherical spots were typical for carotenoids.

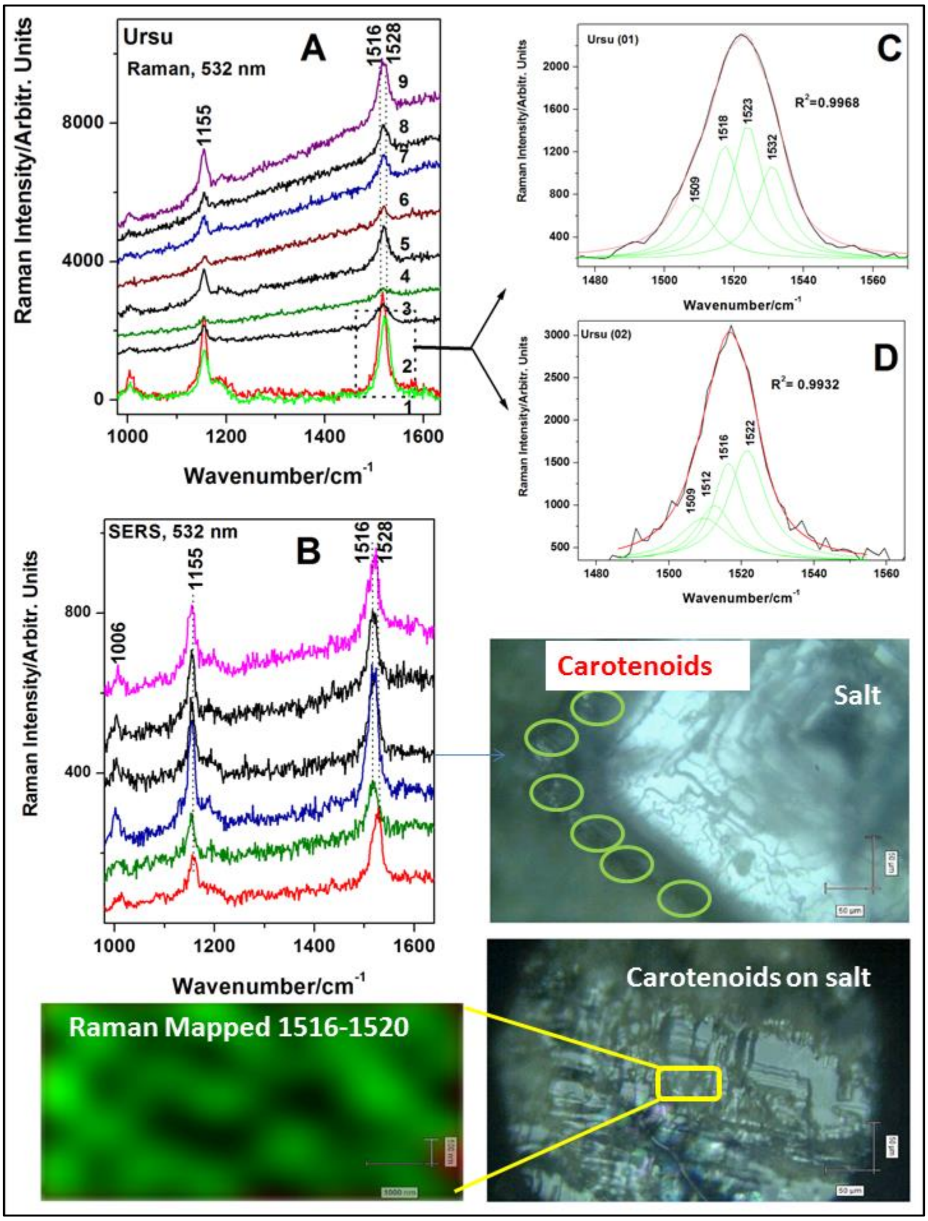

Figure S3. Typical resonance Raman (RR) signal of carotenoids collected from Ursu point samples numbered from 1 to 9. Two distinct signatures are highlighted in spectrum 1 and 2 (A); SERS technique was applied for fluorescence quenching reason revealed similar bands positions but with considerably lower background, decreased absolute intensity under similar collection optics (B); Details of the Lorentzian deconvolution of the main C=C band are showed in C) for spectrum 1 and D) for spectrum 2, respectively. Distinct bands components

determined with a correlation coefficient R2 > 0.99 are presumably associated with distinct carotenoids species comprised in focused microorganisms. Excitation: 532 nm.

F

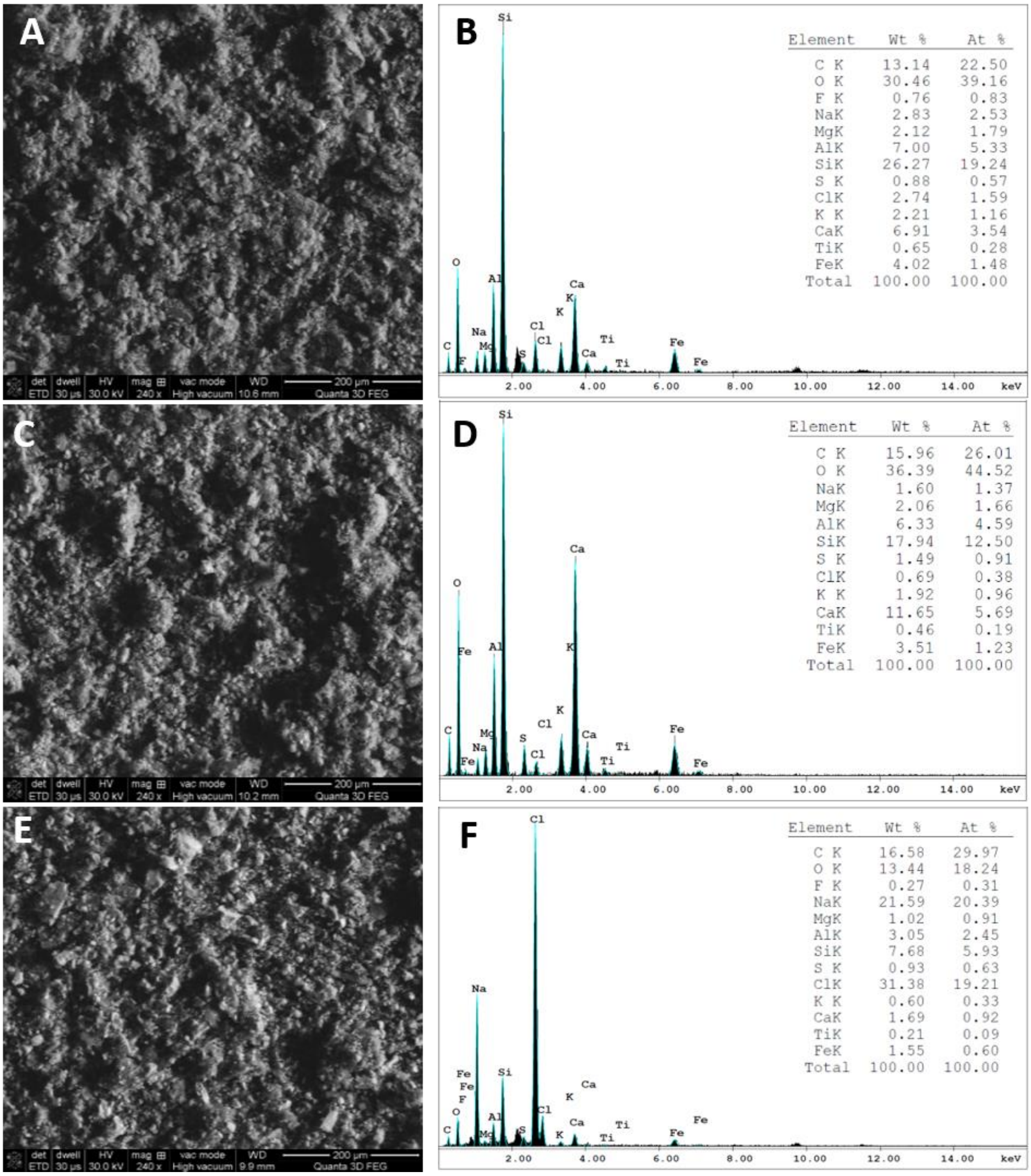

Figure S4. SEM-EDX analysis of Tekirghiol (A, B), Amara (C, D), Ursu (E, F) sapropels.

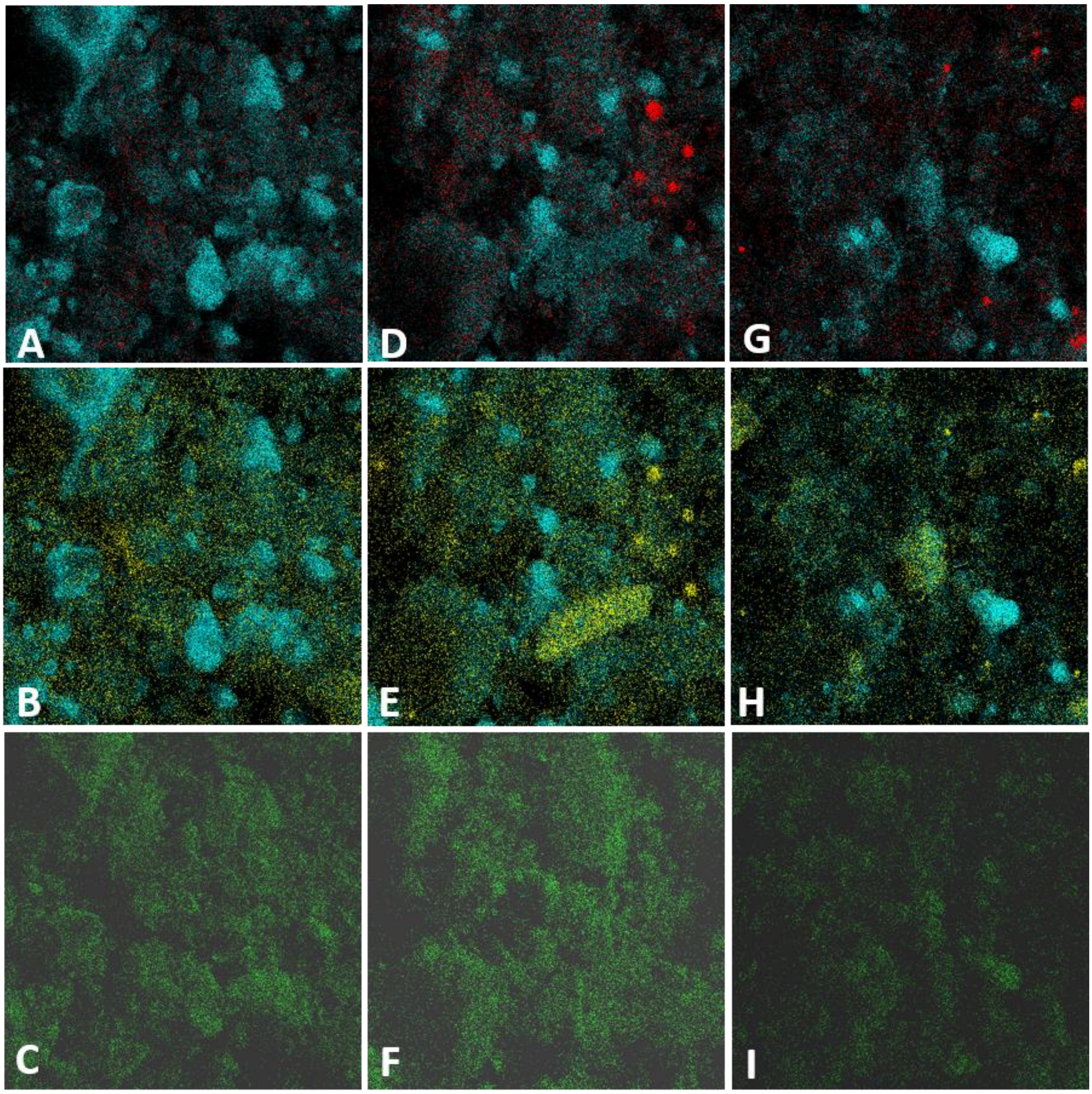

Figure S5. SEM elemental mapping for sulfur (red), iron (yellow), and oxygen (green) in sapropels from Tekirghiol (A, B, C), Amara (D, E, F), and Ursu (G, H, I) lakes. The elemental mapping was performed at x 1000 magnification.

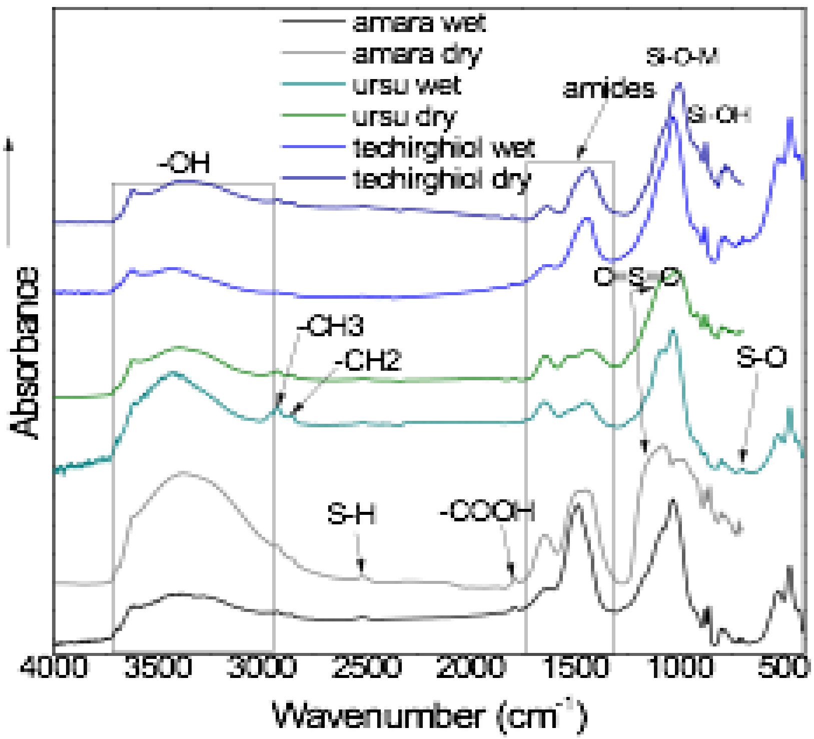

Figure S6. Fourier transform infrared spectroscopy (FT-IR) spectra of sediments samples from Tekirghiol, Amara and Ursu lakes.

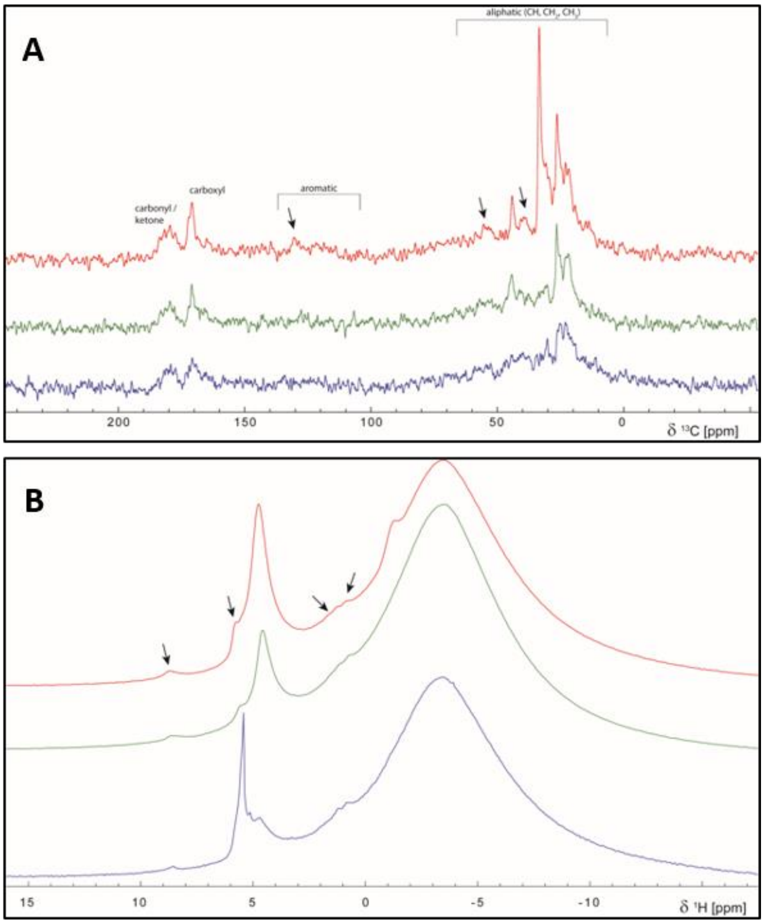

Figure S7. The ^{13}C CP-MAS ss-NMR (A) and ^{1}H ss-NMR spectra (B) recorded on the Tekirghiol (red), Amara (green), and Ursu (blue) lyophilized sapropels. The spectral windows where the ^{13}C NMR lines of various chemical moieties can be typically found are highlighted. By arrows, we mark the positions of very low intensity lines, close to the noise level. The differences between the overall spectral intensities of the samples correlate well with the organic carbon quantitative differences determined by the loss-on-ignition. The ^{1}H ss-NMR spectra are dominated by intense NMR lines at -3.4 ppm and in the 4.5-5.5 ppm spectral region, corresponding to inorganic components, -OH and water molecules in various binding environments. By arrows we mark the positions of very low intensity ^{1}H NMR lines associated with organic matter.

Supplementary Methods

FT-IR spectroscopy and SEM

The FT-IR absorption spectra of the dried and homogenized sediments were recorded in reflection configuration with a Jasco 6000 spectrometer (Jasco Co. Ltd, Japan), at room temperature, in the range 400–4000 cm^{-1} and spectral resolution of 4 cm^{-1} using the KBr pellet technique. The FT-IR spectra of the wet sediments were recorded using a Jasco IRT-5000 FT-IR microscope (Jasco Co. Ltd, Japan) coupled to a Jasco FT-IR-6000 spectrometer in reflection configuration in the range 4000–650 cm^{-1}, at 4 cm^{-1} resolution, using the × 32 Cassegranian objective, imaging a sample area of ~50 × 50 µm. The recorded spectra were smoothed by 5-point Savitzky-Golay function. For the SEM investigations, a FEI Quanta 3D FEG dual beam − in high vacuum mode operating at 30 KV, equipped with an ApolloX SDD Energy Dispersive X-ray (EDX) detector was used. The dried and homogenized samples were examined in a random order using scanning electron microscopy (SEM) at x 70, x 240, x 1000 magnification. On the x 240 images energy spectra were collected for 60 seconds and peak intensities for all elements were recorded.

Solid-state ^{13}C- and ^{1}H-NMR

To assess organic matter fraction extractable by alkaline hydrolysis, the wet sapropel samples were mixed 1:1 (v/v) with 2 M NaOH followed by stirring and subsequent supernatant separation and lyophilization. Then solid-state (ss) $^{13}C(^{1}H)$ NMR spectra of the investigated samples were recorded at 125.73 and 499.99 MHz ^{13}C and ^{1}H Larmor frequencies with an AVANCE III 500 MHz spectrometer (Bruker Optik Gmbh, Germany) operating at room temperature. ^{1}H ss-NMR was performed at 14 kHz spinning frequency, using a simple one-pulse experiment. Variable recycle delay values were used to determine the longitudinal relaxation time of the organic vs inorganic component. The ^{13}C CP-MAS (Cross-Polarization under Magic Angle Spinning) spectra were acquired at 14 kHz spinning frequency, 2 ms contact time, and high-power proton decoupling (100 kHz) under TPPM, by averaging 180k transients with a recycle delay of 3 s. All of the reported ^{13}C and ^{1}H ss-NMR spectra are calibrated relative to the CH3 line in TMS (tetramethylsilane), through an indirect procedure which uses adamantane as an external reference.

Pigment analysis, lipids extraction and GC-MS analysis of fatty acids

Total photosynthetic pigments (chlorophyll a and total carotenoids) were determined by spectrophotometric measurements of methanol extracts, as described by Wetzel and Likens [3]. Total lipids (TLs) were extracted from water and sediment samples using a chloroform/methanol mixture [4]. The fatty acid methyl esters (FAMEs) of the total lipids were prepared by an acid-catalyzed transesterification procedure using 1% sulphuric acid in methanol [5]. The FAMEs were analyzed by gas chromatography−mass spectrometry (GC-MS), according to the procedure of [58]. Identification of FAMEs was achieved by comparing their retention times with those of known standards (Bacterial Acid Methyl Ester, BAME Mix Cat. No. 47080-U, Supelco, Sigma Aldrich) and the resulting mass spectra to those in our database (NIST MS Search 2.0).

References

1. Stojanovic, A., Kogelnig, D., Mitteregger, B., Mader, D., Jirsa, F., Krachler, R., & Krachler, R. (2009). Major and trace element geochemistry of superficial sediments and suspended particulate matter of shallow saline lakes in Eastern Austria. *Geochemistry*, **69**(3), 223-234.
2. Johannesson, K. H. and Zhou, X. (1999). Origin of middle rare earth element enrichments in acid waters of a Canadian High Arctic lake. *Geochim. Cosmochim. Acta*, **63**(1), 153-165.
3. Folch, J., Lees, M., Sloane Stanley, G.H. 1957. A simple method for the isolation and purification of total lipids from animal tissues. *J. Biol. Chem.* **226,** 497-509.
4. Dulf, F. V., Vodnar, D. C., Dulf, E. H., and Pintea, A. 2017. Phenolic compounds, flavonoids, lipids and antioxidant potential of apricot (*Prunus armeniaca* L.) pomace fermented by two filamentous fungal strains in solid state system. *Chem. Cent. J.* **11(1),** 1-10.
5. Dulf, F. V., Vodnar, D. C., Dulf, E. H., Diaconeasa, Z., and Socaciu, C. 2018. Liberation and recovery of phenolic antioxidants and lipids in chokeberry (*Aronia melanocarpa*) pomace by solid-state bioprocessing using *Aspergillus niger* and *Rhizopus oligosporus* strains*. LWT*. **87,** 241-249.
